# Pulmonary‐Targeted Nanoparticles Interrupt the Malignant Mechanical and Biochemical Signaling Crosstalk for Idiopathic Pulmonary Fibrosis Therapy

**DOI:** 10.1002/advs.202512658

**Published:** 2025-11-17

**Authors:** Xue‐Na Li, Ya‐Ping Lin, Xi‐Xi Ma, Yue‐Fei Fang, Hui Wang, Chun‐Hui Cui, Chen Zhang, Jin‐You Piao, Jee‐Heon Jeong, Xian Wu Cheng, Lei Xing, Hu‐Lin Jiang

**Affiliations:** ^1^ Department of Cardiology and Hypertension Affiliated Hospital of Yanbian University Yanji 133000 China; ^2^ College of Pharmacy Yanbian University Yanji 133000 China; ^3^ State Key Laboratory of Natural Medicines Department of Pharmaceutics China Pharmaceutical University Nanjing 210009 China; ^4^ Joint International Research Laboratory of Target Discovery and New Drug Innovation MOE China Pharmaceutical University Nanjing 210009 China; ^5^ Department of Precision Medicine School of Medicine Sungkyunkwan University Suwon 16419 South Korea; ^6^ Department of Endocrinology Zhongda Hospital School of Medicine Southeast University Nanjing 210009 China

**Keywords:** extracellular matrix, idiopathic pulmonary fibrosis, malignant crosstalk, mechanical and biochemical signals, mechanotransduction, transforming growth factor‐beta

## Abstract

Idiopathic pulmonary fibrosis (IPF) involves transforming growth factor‐beta, a key factor that drives biochemical signaling pathways, inducing cellular transdifferentiation and excessive extracellular matrix (ECM) deposition. Increased ECM stiffness alters the mechanical microenvironment of the lung, exacerbating pulmonary dysfunction through mechanical signaling transduction. Here, persistent malignant mechanical and biochemical signaling crosstalk in IPF is demonstrated that drives the relentless progression of the disease. Therefore, inhalable lung‐targeted lipid nanoparticles (VB‐RT NPs) are developed for co‐delivering verteporfin and berbamine to effectively treat IPF by interrupting pulmonary mechanical‐biochemical signaling malignant crosstalk. Specifically, VB‐RT NPs are modified with tannic acid to scavenge reactive oxygen species and enhance lung targeting, and with L‐arginine to penetrate dense ECM and reach deeper lung regions. After being inhaled in a bleomycin model, VB‐RT NPs inhibited fibroblast activation and promoted the transition of endothelial cell (EC)‐like myofibroblasts to ECs, reducing endothelial‐to‐mesenchymal transition and fibrotic progression. Additionally, VB‐RT NPs blocked the nuclear translocation of the mechanotransducers Yes‐associated protein, interrupting fibrosis‐related mechanotransduction pathways. The results demonstrate that VB‐RT NPs effectively reversed dysregulated mechanical‐biochemical signaling crosstalk in fibrotic lungs and halted fibrosis progression, offering a promising therapeutic approach for IPF.

## Graphical abstract

Idiopathic pulmonary fibrosis (IPF) progression is driven by TGF‐β induced biochemical signals and mechanical signals from increased ECM stiffness. Pulmonary‐targeted VB‐RT NPs not only eliminate excessive ROS and penetrate the dense ECM collagen barrier via NO release, but also simultaneously interrupt the persistent malignant between mechanical and biochemical signaling crosstalk, significantly enhancing therapeutic efficacy in IPF treatment.

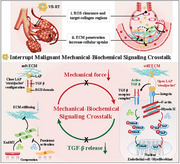

## 1 Introduction

Idiopathic pulmonary fibrosis (IPF) is a progressive lung disorder characterized by excessive extracellular matrix (ECM) deposition, leading to pulmonary tissue fibrosis and irreversible impairment of lung function.^[^ ^1^ ^]^ Currently, IPF remains incurable, with a median survival of only 2–5 years after diagnosis.^[^ ^2^ ^]^ With the aging global population, the burden of IPF on patients and healthcare systems is expected to rise substantially in the coming years.^[^ ^3^, ^4^ ^]^ There is considerable evidence highlighting the critical role of biochemical signaling pathways in driving fibrogenesis.^[^ ^5^, ^6^, ^7^, ^8^ ^]^ In response to fibrotic stimuli, key cytokines such as transforming growth factor‐β (TGF‐β) and tumor necrosis factor‐α are activated, driving epithelial apoptosis, myofibroblast overactivation, and excessive ECM deposition that collectively accelerate fibrosis progression.^[^ ^9^, ^10^, ^11^ ^]^ Meanwhile, the activation of myofibroblasts, accompanied by increased α‐smooth muscle actin (α‐SMA) expression, promotes cytoskeletal remodeling.^[^ ^12^ ^]^ This process alters lung mechanical properties, enhances contractility, and promotes fibrotic collagen deposition and crosslinking, ultimately leading to increased tissue stiffness.^[^ ^13^ ^]^ Therefore, the progression of fibrogenesis is driven by both biochemical and mechanical signaling pathways.

In fact, as a key factor, TGF‐β facilitates the malignant between mechanical and biochemical signals crosstalk, thereby relentlessly promoting disease progression. Specifically, initially secreted in an inactive form into the ECM, TGF‐β1 forms a complex with latency‐associated peptide (LAP) for storage, this latent complex can be stored in the ECM by covalent binding to latent TGF‐β1‐binding protein 1.^[^ ^14^ ^]^ Myofibroblasts sense mechanical forces through actin stress fibers and αv integrins, which transmit these forces to the arginine‐glycine‐aspartate (RGD)‐binding domain in LAP, thereby inducing a conformational change in the latent TGF‐β1 “straitjacket” structure and releasing active TGF‐β1.^[^ ^15^ ^]^ In parallel, damaged endothelial cells (ECs) contribute to fibrosis by releasing vascular endothelial growth factor (VEGF), platelet‐derived growth factor (PDGF), and Jagged‐1, which induce endothelial‐to‐mesenchymal transition (EndMT), enhance ECM production, and reinforce the interplay between mechanical and biochemical signaling.^[^ ^16^, ^17^ ^]^ Perturbations in mechanical forces and matrix stiffness impair endothelial barrier function, fostering EC dysfunction and sustaining fibrotic progression.^[^ ^18^ ^]^ As the disease progresses, mechanical stress on ECs continues to drive metabolic changes that exacerbate fibrosis.^[^ ^19^, ^20^ ^]^ This interplay establishes a vicious cycle. Excessive TGF‐β signaling promotes ECM deposition, enhances mechanosensitivity, and activates downstream effectors such as Yes‐associated protein (YAP) and transcriptional coactivator with PDZ‐binding motif (TAZ), thereby accelerating fibrosis through mechanotransduction.^[^ ^21^ ^]^ Conversely, mechanical cues release latent TGF‐β and stiffen the microenvironment, sustaining fibroblast activation and perpetuating fibrosis even after the resolution of initial inflammation.^[^ ^22^ ^]^ Therefore, targeting the malignant between mechanical and biochemical signaling crosstalk represents a promising therapeutic strategy for IPF.

**Scheme 1:**
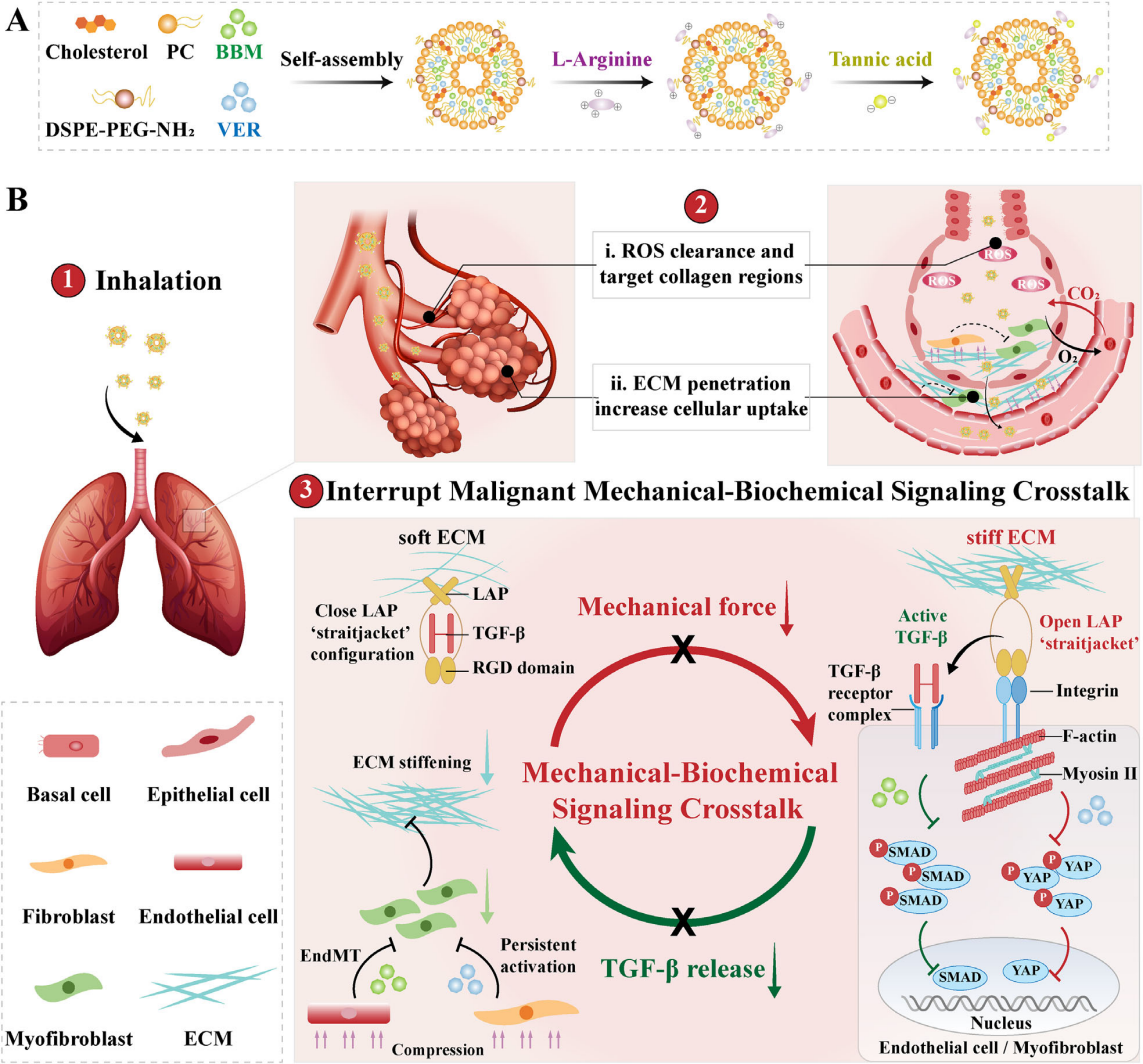
Illustration of pulmonary‐targeted NPs interrupting the malignant mechanical and biochemical signaling crosstalk during IPF therapy. A) Schematic of the preparation of VB‐RT NPs. B) Schematic representation of the in vivo therapeutic process for IPF treatment via pulmonary nebulization of VB‐RT NPs.

In this study, we developed a novel inhalable formulation targeted to the lungs to interrupt the malignant mechanical and biochemical signals crosstalk in the treatment of IPF (**Scheme** **1**). The lipid nanoparticles, termed VB‐RT NPs, were loaded with verteporfin (VER, denoted as V) and berbamine (BBM, denoted as B) co‐modified with L‐arginine (denoted as R) and tannic acid (TA, denoted as T) on the surface of the NPs. The high collagen‐binding affinity of TA promotes targeted accumulation of VB‐RT NPs in lung tissues,^[^ ^23^ ^]^ while its polyphenolic moieties scavenge pulmonary reactive oxygen species (ROS) through interactions with free radicals.^[^ ^24^, ^25^ ^]^ Meanwhile, L‐arginine induces nitric oxide (NO) production in bronchial epithelial cells, which activates endogenous matrix metalloproteinases (MMPs) and promotes ECM degradation, thereby facilitating NP penetration into the lung interstitium and enhancing alveolar delivery.^[^ ^26^, ^27^ ^]^ The combined use of BBM and VER synergistically modulates cellular states in IPF by targeting complementary pathways. BBM upregulates Forkhead box A2 (FOXA2) to inhibit EndMT and restrain fibrosis driven by biochemical signaling,^[^ ^28^ ^]^ whereas VER prevents YAP nuclear translocation and cytoskeletal remodeling, thereby suppressing mechanotransduction.^[^ ^29^ ^]^ By simultaneously disrupting biochemical and mechanical signaling, this combination therapy improves the pulmonary microenvironment and achieves enhanced therapeutic efficacy in IPF.^[^ ^30^ ^]^ This strategy represents an innovative therapeutic approach with strong potential for clinical translation.

## 2 Results

### 2.1 Malignant Mechanical and Biochemical Signaling Crosstalk of the Fibrotic Lung in IPF Patients

**Figure 1:**
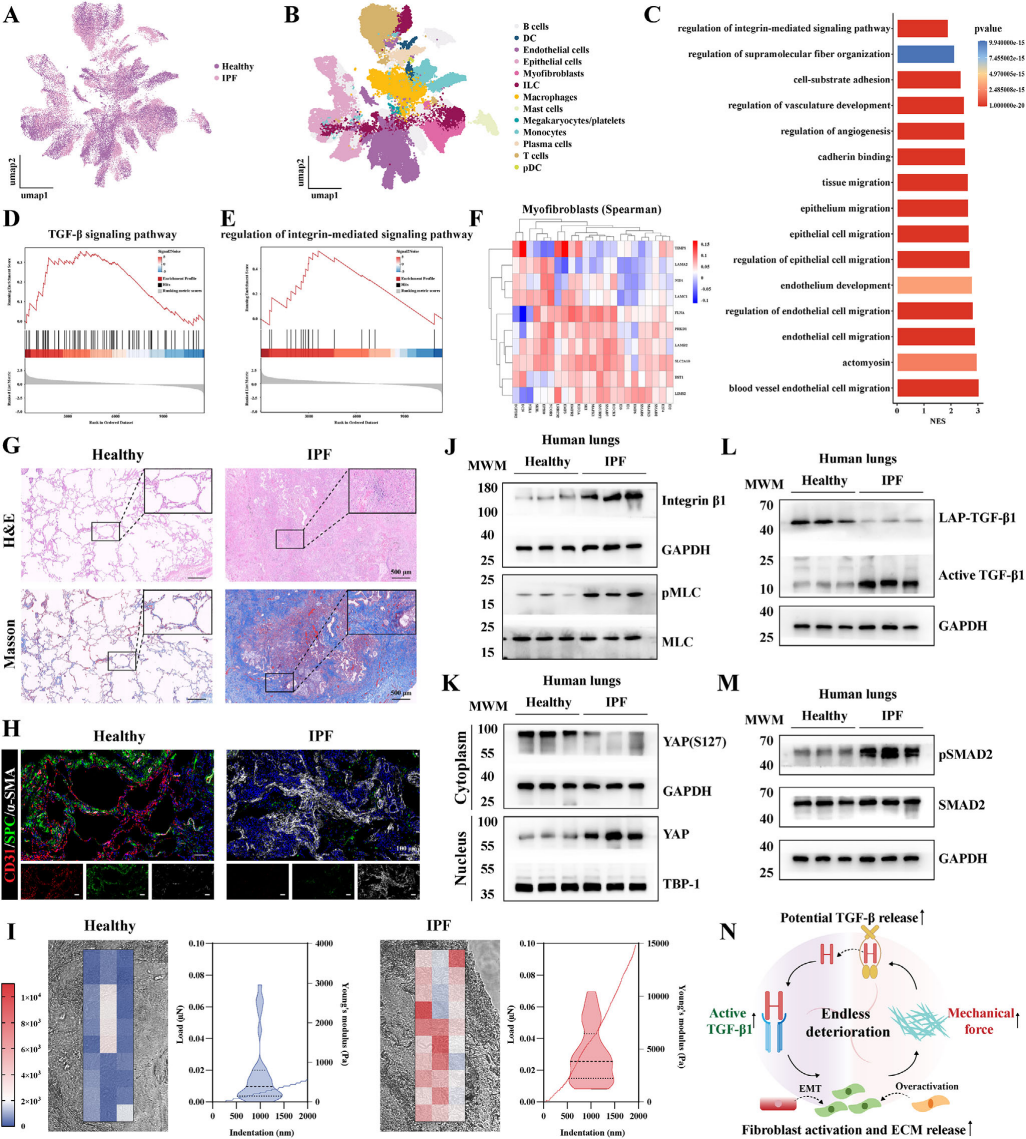
The malignant mechanical‐biochemical signaling crosstalk in samples from healthy donors and IPF patients. A) UMAP distribution of samples from healthy donors and IPF patients. B) UMAP plots of cells from healthy and fibrotic lung datasets colored by cell type. C) GO analyses of differentially expressed genes in fibroblasts from healthy and fibrotic lungs. D) GSEA analysis showed the gene sets of TGF‐β signaling pathway in myofibroblasts. E) GSEA analysis showed the gene sets of regulation of the integrin‐mediated signaling pathway in myofibroblasts. F) Heatmap showing Spearman correlation between integrin and TGF‐β related genes in myofibroblasts. G) H&E staining and Masson's trichrome staining of healthy and fibrotic human lungs. H) Representative IF images of CD31, SPC, and α‐SMA in healthy and fibrotic human lungs. I) Young's modulus of healthy and fibrotic human lungs measured by AFM (n = 3). J) Representative Western blotting (WB) assay of integrin β1, pMLC, and total MLC. K) Representative WB assay of YAP and phosphorylated YAP (S127). L) Representative WB assay of LAP‐TGF‐β1 and Active TGF‐β1. M) Representative WB assay of pSMAD2 and active SMAD2. N) Schematic illustration of malignant mechanical‐biochemical signaling crosstalk in IPF pathogenesis.

To investigate the malignant crosstalk between biochemical and mechanical signaling in fibrotic lungs, we analyzed published single‐cell RNA sequencing data from IPF patients and healthy donors (GSE283885). This analysis identified distinct cellular populations and provided insights into the interrelated signaling pathways driving fibrotic progression (**Figure** **1**A,B). Gene Ontology (GO) analysis showed that fibroblasts were mainly enriched in processes such as integrin‐mediated signaling, cell‐matrix adhesion, vascular development, and EC migration, which are strongly associated with tissue remodeling and fibrosis (Figure 1C). To further assess the involvement of TGF‐β and integrin signaling, Gene Set Enrichment Analysis (GSEA) was performed on ECs and myofibroblasts. The results revealed that TGF‐β and integrin‐associated gene sets were significantly enriched in both cell types, with correlation analysis indicating a strong interconnection between these pathways, consistent with their cooperative activation in fibrotic progression (Figure 1D–F; Figure S1, Supporting Information). Consistently, H&E staining showed progressive fibrotic remodeling accompanied by destruction of alveolar architecture, while Masson's trichrome staining revealed persistently elevated collagen deposition in IPF lungs compared with healthy donors (Figure 1G; Figure S2, Supporting Information). Additionally, immunofluorescence (IF) demonstrated a marked reduction of endothelial (CD31) and alveolar epithelial (Surfactant Protein C, SPC) markers, accompanied by increased α‐SMA expression, reflecting expansion of myofibroblasts and concomitant loss of normal cellular identity (Figure 1H).

Next, we directly evaluated mechanical alterations by measuring the Young's modulus of lung tissue using atomic force microscopy (AFM). Fibrotic lungs exhibited a 6.4‐fold increase in stiffness compared to controls (Figure 1I). At the protein level, Integrin β1 and phosphorylated myosin light chain (pMLC), two key regulators of mechanotransduction, were significantly elevated in fibrotic lungs (Figure 1J). Meanwhile, cytoplasmic phosphorylate YAP (S127) decreased, whereas nuclear YAP increased, indicating aberrant activation of mechanotransduction pathway. (Figure 1K; Figure S3, Supporting Information). Furthermore, IPF lungs showed decreased unprocessed LAP‐TGF‐β along with increased pSMAD expression, which positively correlated with activated TGF‐β (Figure 1L,M). These observations suggest that tissue stiffening facilitates the maturation of latent TGF‐β and amplifies downstream signaling. Taken together, our findings indicate that during IPF progression, elevated mechanical forces promote latent TGF‐β release, activate TGF‐β signaling, and drive EndMT and myofibroblast overactivation. These processes enhance ECM deposition, increase tissue stiffness, and create a self‐reinforcing loop between mechanical and biochemical signaling, driving the progression of IPF (Figure 1N).

### 2.2 Characterization and Multifunctional Properties of the VB‐RT NPs

**Figure 2:**
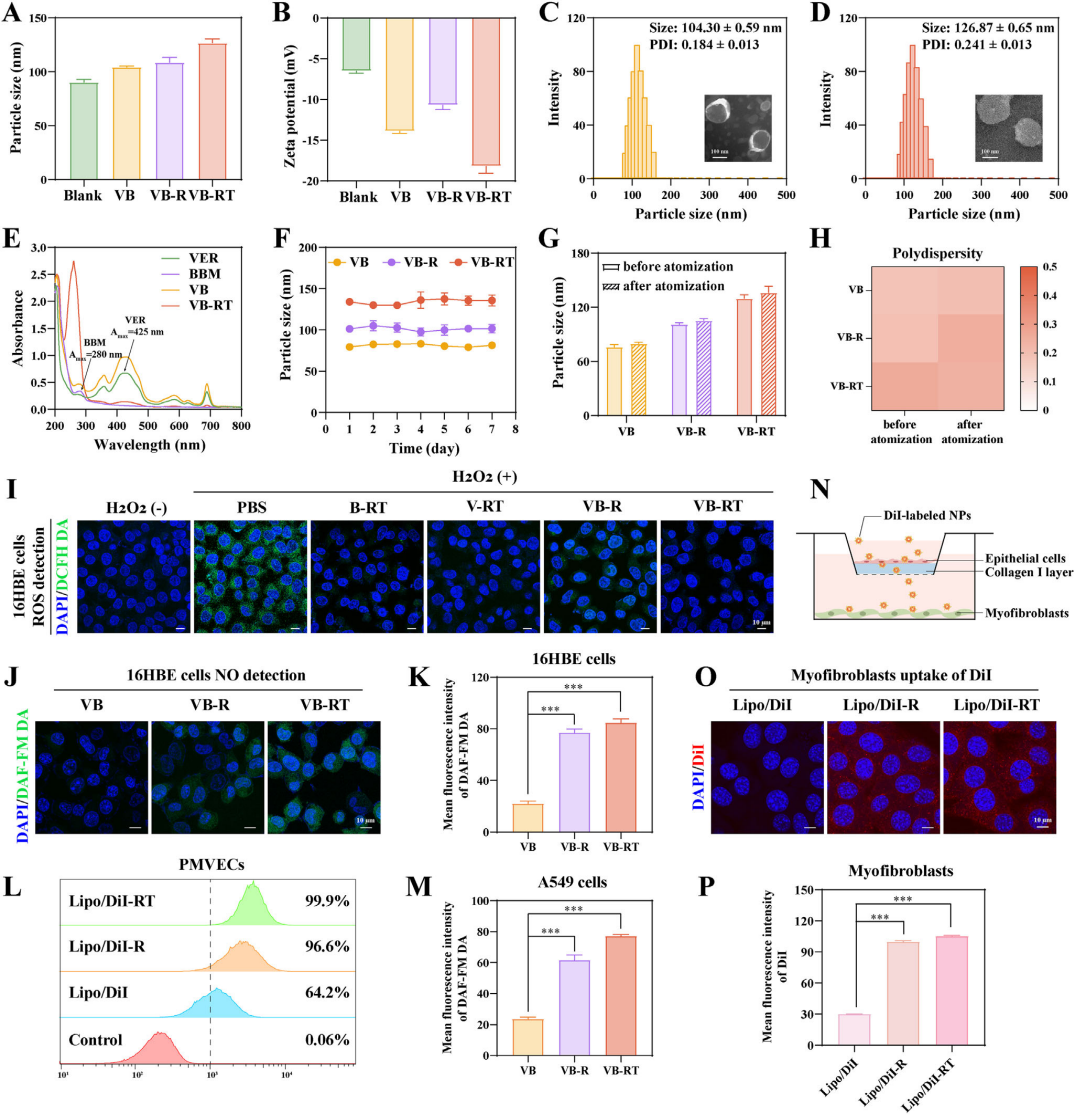
Characterization and multifunctional properties of the VB‐RT NPs. A) Particle size of different formulations. B) Zeta potential of different formulations. C‐D) Average size distribution and TEM images of VB NPs and VB‐RT NPs. E) UV–vis spectra of different formulations across the full wavelength range. F) Seven‐day stability of VB NPs, VB‐R NPs, and VB‐RT NPs. G) Comparison of particle size before and after nebulization. H) Heatmap of PDI before and after nebulization. I) Fluorescence imaging of ROS levels in 16HBE cells under different formulations using DCFH DA. J) The NO detection in 16HBE cells under different formulations using DAF‐FM DA. K) Quantification of mean fluorescence intensity (MFI) of NO in 16HBE cells under different formulations (n = 3). L) Uptake of different formulations in PMVECs measured by flow cytometry. M) Quantification of MFI of NO in A549 cells under different formulations using DAF‐FM DA (n = 3). N) Illustration of the in vitro collagen barrier model, showing DiI‐labeled NPs crossing the epithelial cell‐collagen layer to reach myofibroblasts. O) CLSM images of myofibroblasts treated with different formulations in Transwell chambers with collagen barrier for 4 h at 37 °C. P) Quantification of MFI in myofibroblasts analyzed using ImageJ (n = 3). All data are presented as the Mean ± SD (n = 3). ^***^ *p* < 0.001.

The VB‐RT NPs were constructed by co‐assembling VER and BBM into lipid bilayers, followed by surface modification with L‐arginine and TA.^[^ ^31^, ^32^ ^]^ As shown in Figure 2A, drug encapsulation and surface modification resulted in a gradual increase in particle size, with VB NPs measuring 104.30 ± 0.59 nm and VB‐RT NPs showing a larger size of 126.87 ± 0.65 nm. Consistently, Zeta potential analysis revealed that the introduction of positively charged L‐arginine slightly increased the potential of VB‐RT NPs compared with VB NPs, whereas further modification with TA reduced the Zeta potential to −18.19 ± 0.87 mV, a range favorable for traversing the negatively charged tracheal mucus layer (Figure 2B). Transmission electron microscopy confirmed that both VB NPs and VB‐RT NPs exhibited uniform spherical morphologies (Figures 2C,D). In addition, the UV‐Vis spectra displayed characteristic absorption peaks corresponding to VER and BBM, confirming the successful encapsulation of both drugs in the formulations (Figure 2E). The disappearance of the BBM absorption band in VB‐RT NPs mainly results from the strong UV absorption of the TA coating, whose spectrally overlaps with BBM and masks its signal. Quantitative analysis by HPLC further demonstrated high encapsulation efficiency (EE) and loading capacity (LC) for VER, with VB NPs reaching an EE of 99.04 ± 0.78% and LC of 1.67 ± 0.06%, while VB‐RT NPs maintained an EE of 95.91 ± 1.05% and LC of 0.76 ± 0.01% (Figure S4, Supporting Information). Moreover, colorimetric and Sakaguchi assays verified the successful grafting of TA and L‐arginine, with rates of 0.88 ± 0.02% and 1.56 ± 0.04%, respectively, confirming efficient surface modification of VB‐RT NPs (Figure S5, Supporting Information). We next assessed the stability and aerosolization performance of the formulations. Over 7 days of monitoring, all formulations exhibited good colloidal stability (Figure 2F). Notably, both the particle size and polydispersity index (PDI) remained unchanged before and after nebulization, indicating that these NPs are structurally stable and suitable for aerosol delivery (Figures 2G,H).

The ROS‐scavenging ability of VB‐RT NPs was evaluated using the DCFH‐DA fluorescent probe. Compared with the control group, H_2_O_2_‐pretreated 16HBE cells displayed strong green fluorescence, whereas VB‐RT NPs treatment markedly suppressed ROS generation (Figures 2I, S6, Supporting Information). Consistent results were observed in A549 cells (Figure S7, Supporting Information). The administration concentration of VB‐RT NPs was selected based on in vitro cytotoxicity assays (Figure S8, Supporting Information). Furthermore, VB‐RT NPs exhibited significantly stronger inhibitory effects on fibroblasts compared with single‐drug formulations. The combination index (CI), calculated by the Chou‐Talalay method, remained consistently > 1 across multiple effect levels (IC_25_IC_90_), confirming a synergistic interaction rather than a merely additive effect. These findings substantiate the rationale for employing VB‐RT NPs as a combination strategy (Figure S9, Supporting Information). We investigated NO generation in epithelial cells using the DAF‐FM DA probe. In 16HBE cells, L‐arginine‐modified formulations, including VB‐R NPs and VB‐RT NPs, induced a significant increase in NO production (Figures 2J, K). Consistent results were observed in A549 cells, where NO levels were similarly enhanced by both formulations (Figures 2M and S10, Supporting Information). In pulmonary microvascular endothelial cells (PMVECs), Lipo/DiI‐RT NPs exhibited markedly higher cellular uptake compared with Lipo/DiI NPs, and similar results were observed in myofibroblasts with Lipo/C6‐RT NPs (Figures 2L, S11 and S12, Supporting Information). The enhanced uptake of L‐arginine and TA‐modified NPs can be attributed to several factors, including the absence of ECM barriers that permit direct membrane interaction, L‐arginine promoting NO production to increase membrane fluidity,^[^ ^33^ ^]^ and TA improving NP stability and enhancing membrane interaction.^[^ ^34^ ^]^

To directly verify the ECM penetration efficiency of different NPs, we established a Transwell chamber model (Figure 2N). The 16HBE cells were seeded in the collagen‐coated upper chamber, while myofibroblasts were pre‐seeded in the lower chamber. We assessed the NO‐mediated mechanism by measuring MMP‐2/9 expression, FITC‐collagen degradation, and myofibroblast uptake of DiI‐labeled NPs. Compared with the Lipo/DiI group, both Lipo/DiI‐R and Lipo/DiI‐RT significantly upregulated MMP‐2/9 expression (Figure S13, Supporting Information), accompanied by reduced green fluorescence of the collagen matrix, indicating enhanced ECM degradation (Figure S14, Supporting Information). Consistently, myofibroblasts in the lower chamber showed the strongest red fluorescence in the Lipo/DiI‐RT group, confirming superior penetration and cellular uptake (Figures 2O, P). These findings demonstrate that L‐arginine modification promotes NO production, which activates MMPs to degrade ECM and thereby enhances Lipo/DiI‐RT NPs penetration and uptake. To further validate the roles of ROS and NO in NP penetration, we performed intervention experiments using NAC (ROS scavenger) and L‐NAME (NOS inhibitor) in a Transwell model. Lipo/DiI‐RT showed markedly improved ECM penetration and fibroblast uptake compared with unmodified NPs, but this effect was reduced by L‐NAME, confirming the importance of NO. Under elevated ROS conditions (H_2_O_2_ treatment), TA scavenging of ROS increased NP uptake, while NAC further restored ECM permeability. Excessive ROS densified ECM through collagen crosslinking and disrupted epithelial integrity, thereby reinforcing the barrier, whereas NAC alleviated these effects. Notably, the combination of NAC and L‐NAME again suppressed penetration, indicating that both ROS clearance and NO production are indispensable (Figure S15, Supporting Information). In addition, we evaluated the delivery of modified NPs in the presence of a mucus barrier, and the results showed that they were effectively internalized by 16HBE cells (Figure S16, Supporting Information). Collectively, these findings demonstrate that dual modification with L‐arginine and TA markedly enhances NPs uptake and delivery efficiency, thereby providing a solid foundation for subsequent therapeutic applications.

### 2.3 VB‐RT NPs Interrupt Malignant Mechanical and Biochemical Signaling Crosstalk in Fibroblasts In Vitro

**Figure 3:**
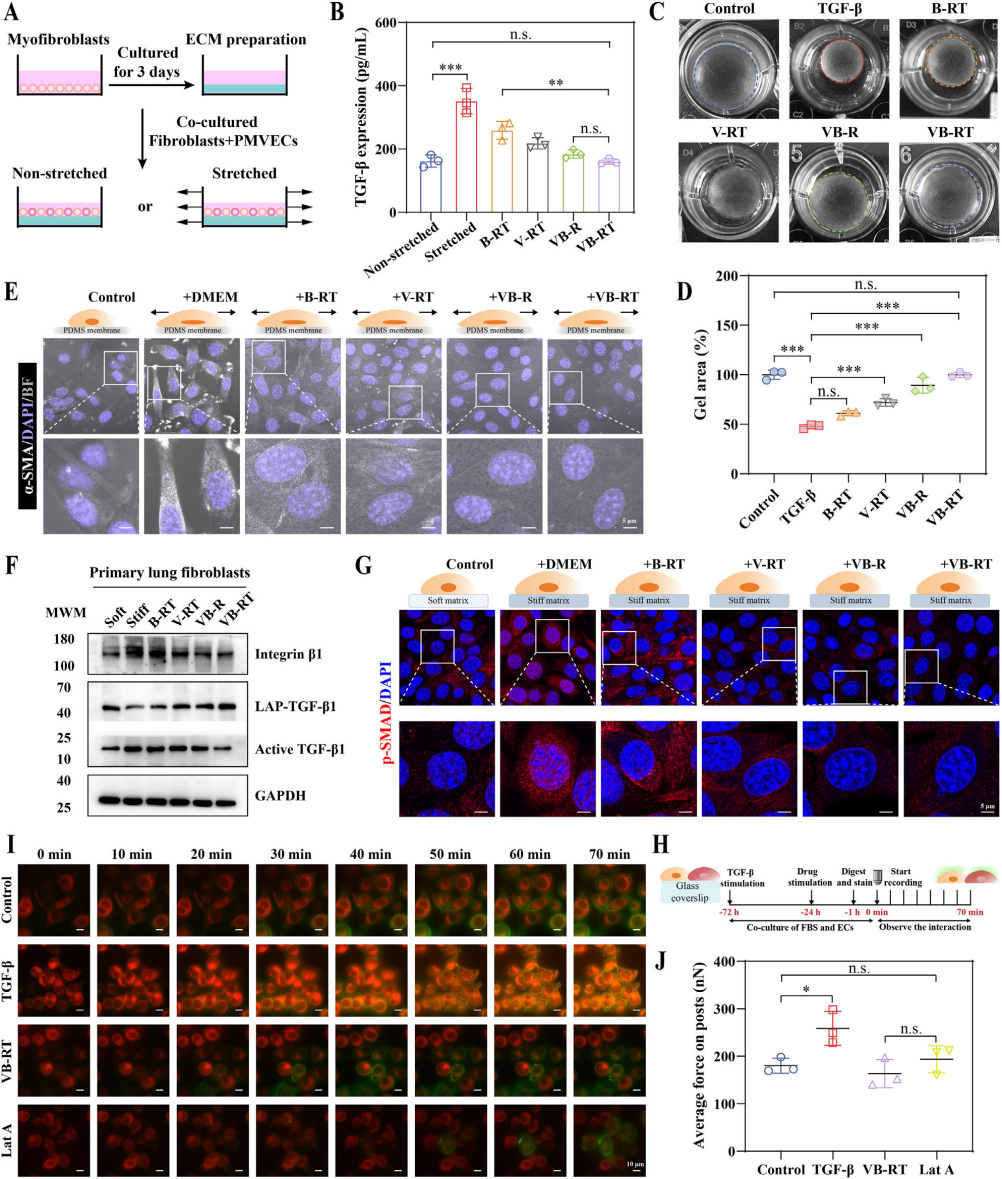
VB‐RT NPs interrupt malignant mechanical and biochemical signaling crosstalk in fibroblasts in vitro. A) Schematic illustration of potential TGF‐β release from the ECM. B) The TGF‐β expression levels of fibroblasts and PMVECs were cultured on non‐stretched and stretched membranes (n = 3). C) Representative images of collagen gel contraction mediated by fibroblasts after treatment with different formulations. D) Quantitative analysis of gel area contraction (n = 3). E) IF staining of α‐SMA (white) and DAPI (purple) in fibroblasts subjected to cyclic stretching or static culture using the programmable mechanical cell stretch system. F) Representative WB assay of integrin β1, LAP‐TGF‐β1, and active TGF‐β1. G) IF staining of p‐SMAD (red) and DAPI (blue) in fibroblasts cultured on soft and stiff matrices. H) Schematic of live cell imaging of fibroblasts and PMVECs. I) Imaging of fibroblast‐PMVEC contact (1 frame/10 min). J) Quantification of average force on micropillars by fibroblasts under different conditions (n = 3). ^*^ *p* < 0.05, ^**^ *p* < 0.01, and ^***^ *p* < 0.001. n.s., no significant difference.

The mechanical activation of latent TGF‐β in the ECM was investigated using stretchable culture chambers seeded with myofibroblasts. After three days, the myofibroblasts were removed, and fibroblasts and PMVECs were co‐cultured on the preconditioned ECM (**Figure** **3**A). Quantification of TGF‐β demonstrated that mechanical stretching significantly enhanced activation of latent TGF‐β, yielding a 2.16‐fold increase compared with non‐stretched controls. Importantly, VB‐RT NPs treatment reduced the release of active TGF‐β, restoring levels to baseline comparable to those observed prior to stretching (Figure 3B). To assess whether formulation could suppress the acquisition of a contractile phenotype, we employed a floating collagen gel contraction assay, in which gel surface area inversely reflects cellular contractile force. TGF‐β stimulation markedly enhanced collagen contraction, an effect attenuated by all treatment groups, with the most significant reversal observed in the VB‐RT NPs‐treated group. These results indicate that VB‐RT NPs effectively mitigate TGF‐β‐induced contractility (Figure 3C). Quantitative analysis of gel surface area further confirmed these findings (Figure 3D). Similarly, under cyclic mechanical stretching, fibroblasts displayed intense α‐SMA fluorescence, which was reduced in treatment groups, with VB‐RT NPs showing the most effective inhibition of fibroblast activation (Figure 3E; Figure S17, Supporting Information).

Next, we compared fibroblast behavior on soft and stiff matrices. The Young's modulus of the matrices was 2.42 ± 0.69 kPa for soft and 8.52 ± 1.02 kPa for stiff substrates (Figure S18, Supporting Information). On a stiff substrate, integrin β1 in fibroblasts was activated, and this phenomenon was attenuated to varying degrees by different treatment groups (Figure 3F; Figure S19, Supporting Information). WB analysis revealed that fibroblasts on stiff matrices exhibited pronounced activation of latent TGF‐β and increased conversion to its active form. All treatments suppressed this effect, with VB‐RT NPs exerting the strongest inhibition (Figure 3F; Figure S20, Supporting Information). Consistently, fibroblasts on stiff substrates exhibited elevated SMAD phosphorylation, a key downstream signaling event. IF staining revealed intense red fluorescence and nuclear localization in fibroblasts cultured on stiff ECM, while VB‐RT NPs significantly reduced fluorescence intensity and inhibited SMAD nuclear translocation (Figure 3G). Quantification of nuclear p‐SMAD‐positive cells further corroborated this finding (Figure S21, Supporting Information). These findings indicate that VB‐RT NPs effectively disrupt the malignant feedback loop between mechanical strain and biochemical TGF‐β signaling in fibroblasts. To further investigate the interaction between fibroblasts and ECs in a fibrotic environment, fibroblasts and ECs were cultured on glass‐bottom dishes and stained for cell morphology and stress fibers (Figure 3H). Following VB‐RT NPs treatment, F‐actin intensity was significantly reduced, and cell‐cell adhesion weakened. Comparable results were observed with the actin polymerization inhibitor Latrunculin A (Lat A), which diminished F‐actin expression between cells (Figure 3I). These results suggest that VB‐RT NPs mitigate fibrotic remodeling by weakening fibroblast‐EC interactions. To directly assess cellular forces, traction‐force microscopy on micropillar substrates showed that TGF‐β‐stimulated myofibroblasts generated contractile forces localized at adhesion points, with vectors directed toward the cell center (Figure S22, Supporting Information). Treatment groups reduced these traction forces, with VB‐RT NPs producing effects comparable to Lat A (Figure 3J). These findings suggest that VB‐RT NPs may influence the fibrotic process by reducing intercellular forces and interactions.

### 2.4 VB‐RT NPs Interrupt Malignant Mechanical and Biochemical Signaling Crosstalk in ECs In Vitro

**Figure 4:**
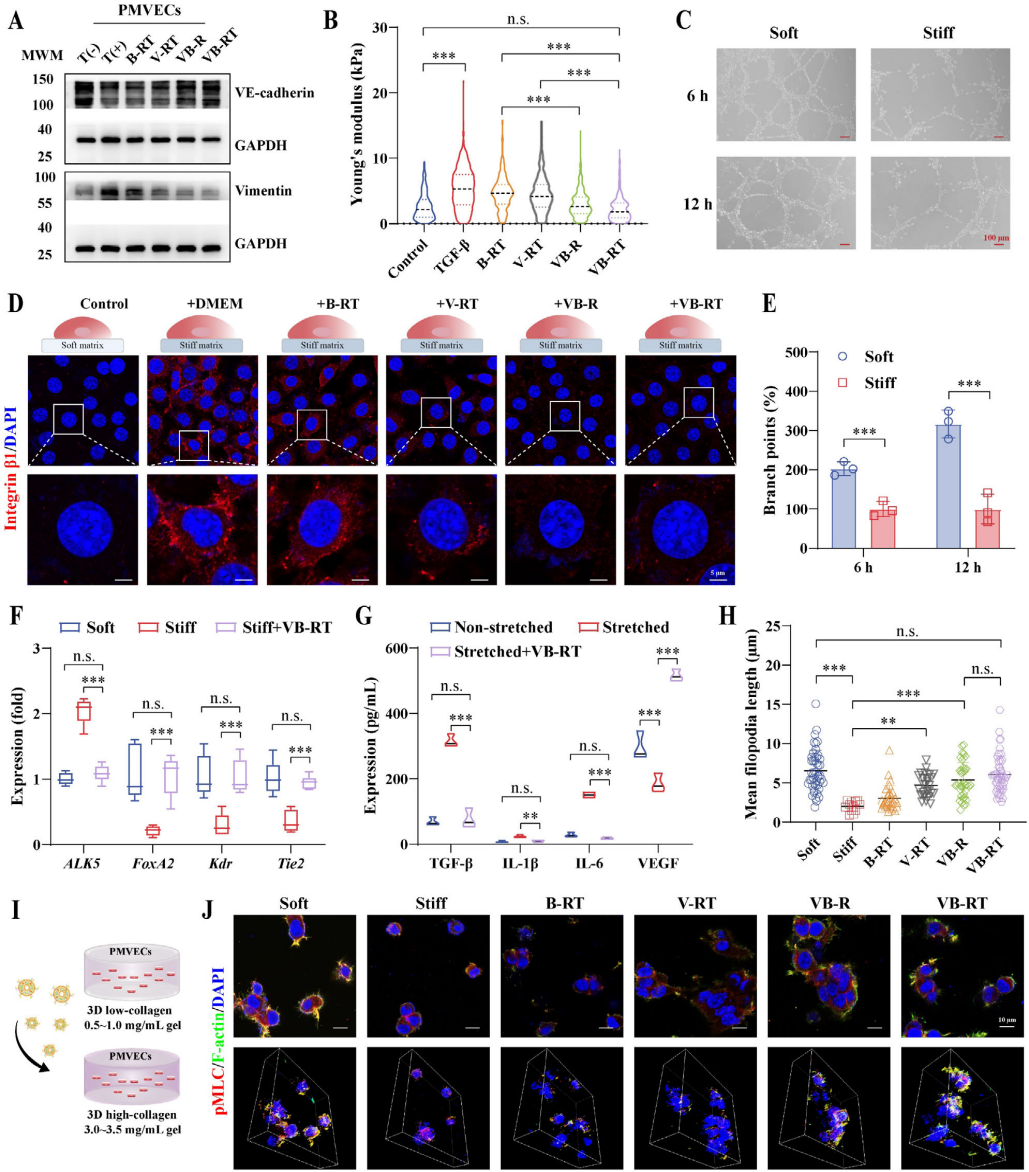
VB‐RT NPs interrupt malignant mechanical and biochemical signaling crosstalk in ECs in vitro. A) Representative WB assay of VE‐cadherin and Vimentin. B) Young's modulus of PMVECs measured by AFM indentation after treatment with different formulations (n = 200 measurements per cell). C) Representative images of tube formation on soft and stiff matrices at 6 and 12 h. D) IF staining of integrin β1 (red) and DAPI (blue) in fibroblasts cultured on soft and stiff matrices. E) Quantification of branch points in the tube formation on soft and stiff matrices at 6 and 12 h (n = 3). F) Quantitative PCR analysis of gene expression (*ALK5*, *FoxA2*, *Kdr*, *Tie2*) under different treatments (n = 6). G) ELISA quantification of cytokine levels (TGF‐β, IL‐1β, IL‐6, VEGF) under non‐stretched, stretched, and VB‐RT NPs conditions (n = 3). H) Quantitative analysis of filopodia length after 24 h in encapsulated EC spheroids cultured on soft and stiff matrices (from left to right n = 6, 6, 8, 8, 8, 8 cells). I) Schematic illustration of 3D cell culture in soft‐collagen (0.5–1.0 mg mL^−1^) and stiff‐collagen (3.0–3.5 mg mL^−1^) gels. J) IF staining of pMLC (red), F‐actin (green), and DAPI (blue) in cells cultured in 3D gels with soft and stiff conditions. The top images show 2D views, while the bottom images display 3D projections. ^**^ *p* < 0.01, and ^***^ *p* < 0.001. n.s., no significant difference.

As shown in the following analysis, TGF‐β stimulation upregulated Vimentin and downregulated vascular endothelial cadherin (VE‐cadherin), indicating endothelial activation and EndMT induction. Treatment effectively attenuated these changes, with VB‐RT NPs demonstrating the strongest suppression of EndMT in PMVECs (**Figure** **4**A; Figure S23, Supporting Information). Next, we assessed morphological and biomechanical alterations in ECs. Compared with controls, PMVECs exposed to TGF‐β exhibited a marked increase in Young's modulus, reflecting elevated stiffness. All treatment groups reduced stiffness, with the most substantial decrease observed in VB‐RT NPs‐treated cells (Figure 4B). These findings suggest that VB‐RT NPs mitigate the mechanical stress associated with mesenchymal transition, thereby contributing to the restoration of a healthier microenvironment. We further investigated the proangiogenic potential of NPs using tube formation assays on substrates of different stiffness. Notably, stiff matrices markedly reduced both tube length and branching points (Figure 4C,E; Figure S24, Supporting Information). By contrast, VB‐RT NPs significantly improved angiogenesis, increasing both parameters compared with single‐drug or unmodified groups (Figure S25, Supporting Information). Consistently, gene expression analysis showed that *Tie2* and *Kdr* were downregulated in ECs cultured on stiff matrices but were restored to baseline levels after VB‐RT NPs treatment (Figure 4F). We also investigated transcription factors associated with endothelial differentiation. Under stiff substrate conditions, *ALK5* was upregulated while *FoxA2* was suppressed. Treatment with VB‐RT NPs reversed these changes by promoting *FoxA2* expression and inhibiting *ALK5*. These findings suggest that VB‐RT NPs redirect EC differentiation from myofibroblast‐like phenotypes toward an endothelial identity. In parallel, cytokine analysis revealed that mechanical stretching of PMVECs increased secretion of TGF‐β, IL‐1β, and IL‐6, whereas VB‐RT NPs reduced their release. Importantly, VEGF expression, suppressed under fibrotic conditions, was significantly restored, indicating preserved endothelial function and enhanced angiogenesis (Figure 4G).

To delineate the pathways by which ECs sense mechanical cues, we next examined integrin and cytoskeletal dynamics. IF analysis showed that integrin β1 aggregation increased with substrate stiffness and was markedly reduced following treatment (Figure 4D; Figure S26, Supporting Information). To examine the effect of mechanical forces on EC generation, we embedded PMVEC multicellular spheroids in collagen gels of varying stiffness and observed prominent filopodia formation in ECs over 24 h (Figure 4I). In stiff gels, p‐MLC expression was markedly elevated compared with that in soft gels (Figure 4J). As tip cells initiate angiogenesis, EC spheroid budding was used as a proxy for tip cell formation.^[^ ^35^ ^]^ Consistently, budding was reduced in stiff gels but significantly enhanced after VB‐RT NP treatment (Figure S27, Supporting Information). Quantification of filopodia length revealed shorter extensions in stiff gels compared to soft gels, whereas VB‐RT NPs treatment restored filopodia elongation and invasion distance to levels similar to those in soft‐matrix conditions (Figure 4H). These results suggest that VB‐RT NPs can restore the balance between adhesion and contraction in stiff IPF lung environments, thereby facilitating directed EC migration and vascular stabilization.

### 2.5 VB‐RT NPs Biodistribution in the Lungs of Bleomycin (BLM)‐Induced Fibrotic Mice

**Figure 5:**
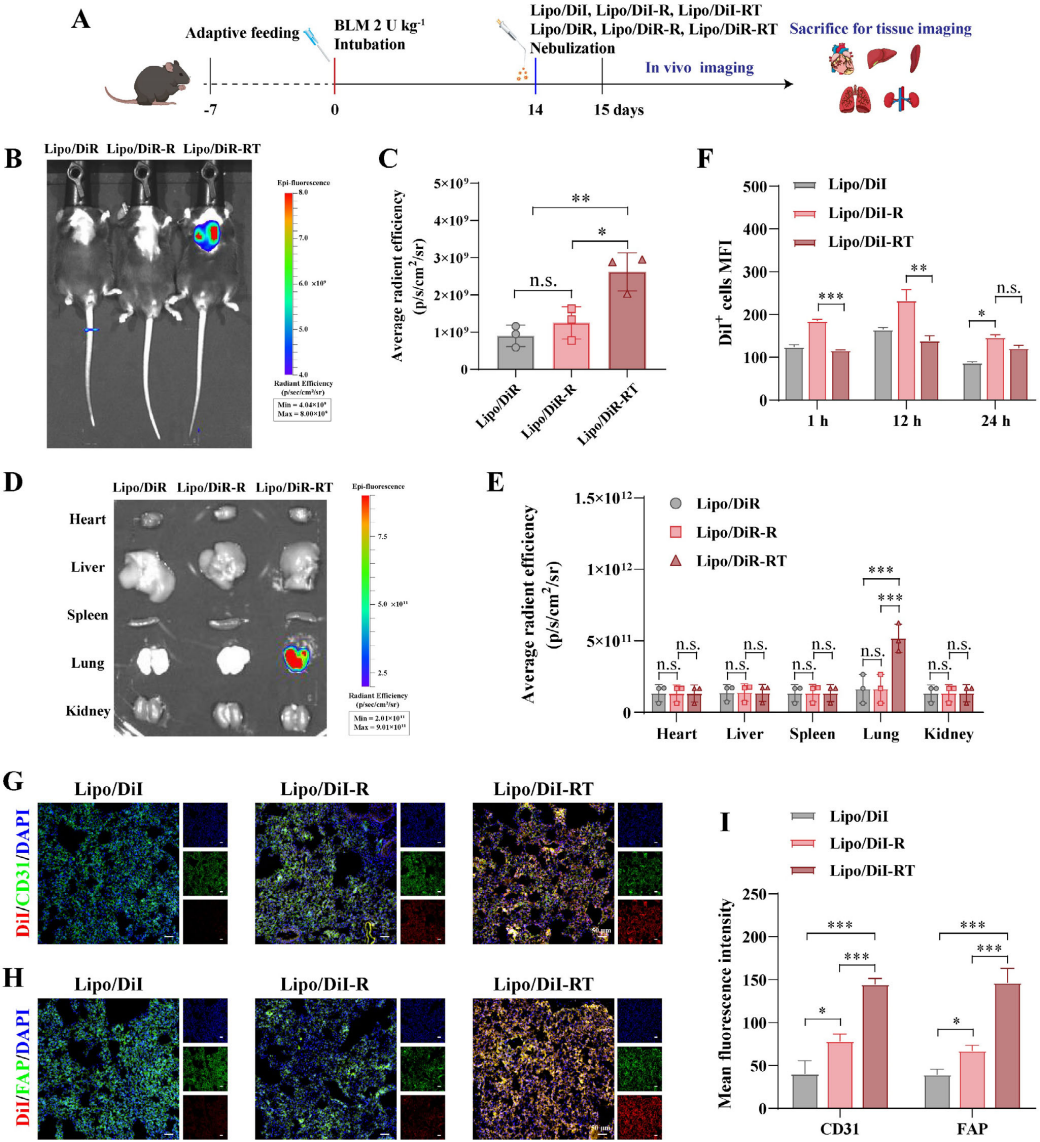
VB‐RT NPs biodistribution in the lungs of BLM‐induced fibrosis mice. A) Schematic illustration of in vivo and ex vivo imaging in the BLM‐induced pulmonary fibrosis model. The red and blue lines indicate the start of model induction and drug administration, respectively. B) In vivo images of mice nebulized with Lipo/DiR, Lipo/DiR‐R, and Lipo/DiR‐RT for 24 h. C) Quantification of fluorescence intensity in the lungs following treatment (n = 3). D) Ex vivo fluorescence images of the heart, liver, spleen, lungs, and kidneys of treated mice. E) Quantitative analysis of fluorescence intensity in major organs following treatment (n = 3). F) MFI of DiI^+^ cells in BALF measured by flow cytometry (n = 3). G and H) Colocalization of DiI‐labeled NPs with CD31 (G) and FAP (H) in the lungs of fibrotic mice. I) Quantification of fluorescence co‐localization with CD31 and FAP expression using ImageJ (n = 3). All data are presented as the Mean ± SD (n = 3). ^*^ *p* < 0.05, ^**^ *p* < 0.01, and ^***^ *p* < 0.001. n.s., no significant difference.

The efficient accumulation and retention of functional NPs at the lesion site is essential for achieving optimal therapeutic efficacy. DiR was encapsulated in lipid NPs with different surface modifications to visualize their distribution, which was tracked using in vivo imaging. As shown in **Figure** **5**A, the IPF model was established in male C57BL/6 mice by a single intratracheal administration of 2 U kg^−1^ BLM and confirmed after 14 days. The Lipo/DiR‐RT group exhibited the highest fluorescence intensity in the lungs, as revealed by in vivo fluorescence imaging 24 h after nebulized administration (Figure 5B,C). These data indicate that dual modification with L‐arginine and TA enhances pulmonary retention and reduces degradation of NPs. To minimize variability caused by tissue depth, major organs were harvested 24 h post‐administration. Consistently, the Lipo/DiR‐RT group showed significantly higher pulmonary fluorescence intensity than Lipo/DiR‐R or Lipo/DiR, in agreement with the in vivo results (Figure 5D,E). This enhancement may result from L‐arginine facilitating NPs penetration through the pathological ECM barrier, while the polyhydroxy structure of TA contributes to prolonged pulmonary retention.

Bronchoalveolar lavage fluid (BALF) is known to consist predominantly of alveolar macrophages (AMs), which also increase in severe COVID‐19 patients.^[^ ^36^ ^]^ We next evaluated pulmonary penetration using Lipo/DiI‐RT in IPF mice. Flow‐cytometric analysis of DiI‐positive BALF cells revealed more efficient uptake of Lipo/DiI‐RT within 12 h, though its fluorescence declined by 24 h, likely due to the enhanced uptake facilitated by NO generated from L‐arginine modification, which also promotes rapid clearance. In contrast, Lipo/DiI‐RT exhibited lower fluorescence in BALF cells at 24 h, suggesting that TA modification reduces AM capture and facilitates deeper penetration into alveolar regions (Figure 5F). Moreover, quantification of DiI in BALF supernatant confirmed greater early release with Lipo/DiI and Lipo/DiI‐R compared to Lipo/DiI‐RT (Figure S28, Supporting Information). Notably, Lipo/DiI‐RT not only evaded excessive AM uptake but also supported rapid drug release into deeper alveolar compartments, thereby improving pulmonary penetration and targeting efficiency. Furthermore, the pulmonary distribution of DiI‐labeled NPs was assessed in lung tissue sections using confocal microscopy. As shown in Figure 5G,I, Lipo/DiI‐RT exhibited substantial co‐localization with CD31‐positive ECs after nebulized delivery, in contrast to unmodified NPs. Moreover, Lipo/DiI‐RT fluorescence overlapped with FAP (fibroblast activation protein)‐positive myofibroblasts, indicating targeted localization to fibrotic regions (Figure 5H,I). In summary, these findings demonstrate that Lipo/DiI‐RT, through dual functional modification, achieves enhanced accumulation and retention in damaged ECs and activated myofibroblasts, which may contribute to improved therapeutic outcomes in IPF.

### 2.6 VB‐RT NPs Modulate Biochemical Signaling and Alleviate Pulmonary Fibrosis in BLM‐Induced Fibrotic Mice

**Figure 6:**
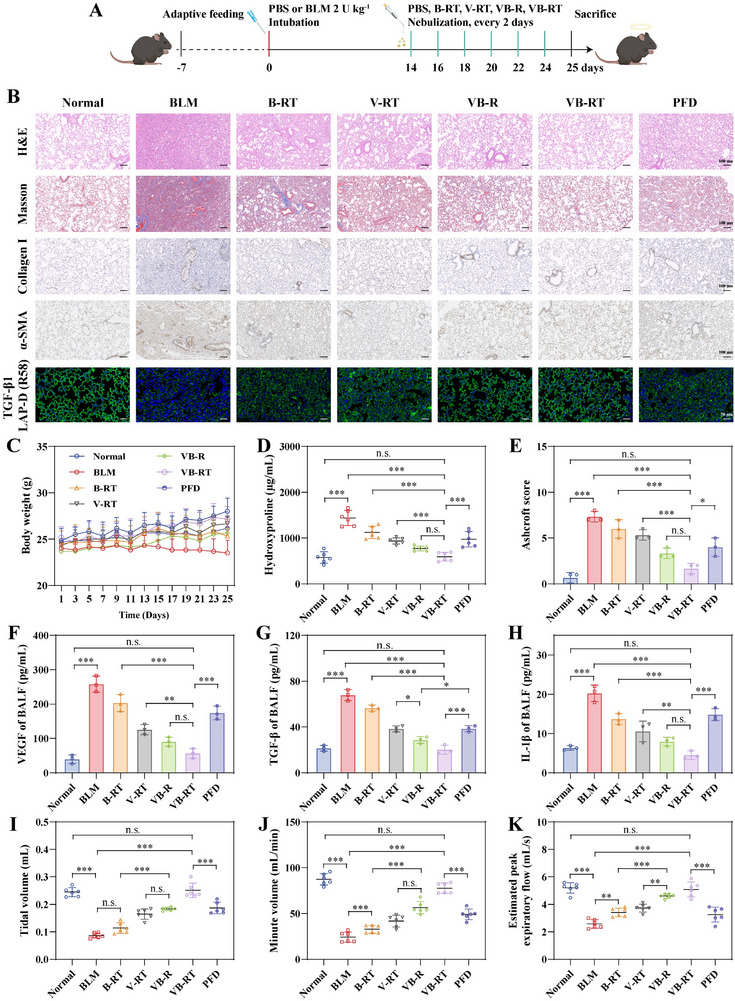
VB‐RT NPs modulate biochemical signaling and alleviate pulmonary fibrosis in BLM‐induced fibrotic mice. A) Schematic of experimental design for therapeutic assessment in BLM‐induced mice. The red and green lines indicate the start of model induction and drug administration, respectively. B) Representative histological analysis of lung sections, including H&E, Masson's trichrome, and IHC staining for α‐SMA and Collagen I, and IF analysis for TGF‐β1 LAP‐D (R58). C) Body weight changes following treatment with different formulations (n = 6). D) The levels of HYP content in lung tissue (n = 6). E) Ashcroft scores of lung fibrosis in normal and BLM‐treated mice (n = 3). F‐H) The levels of VEGF (F), TGF‐β (G), and IL‐1β (H) in BALF, measured by ELISA (n = 3). I‐K) Pulmonary function tests: tidal volume (I), minute volume (J), and peak expiratory flow (K) (n = 6). All data are presented as the Mean ± SD. ^*^ *p* < 0.05, ^**^ *p* < 0.01, and ^***^ *p* < 0.001. n.s., no significant difference.

The anti‐fibrotic efficacy of VB‐RT NPs was evaluated in vivo. As shown in **Figure** **6**A, pulmonary fibrosis was induced in C57BL/6 mice via intratracheal instillation of BLM (2 U kg^−1^). After 14 days, mice were randomly divided into six groups and treated via pulmonary nebulization with physiological saline, B‐RT NPs (BBM‐loaded NPs modified with L‐arginine and TA), V‐RT NPs (VER‐loaded NPs modified with L‐arginine and TA), VB‐R NPs, VB‐RT NPs, or oral pirfenidone (PFD) every other day for a total of six treatments. H&E staining revealed normal alveolar architecture in healthy controls, whereas BLM‐treated mice displayed pronounced alveolar wall thickening, structural disruption, and perivascular fibrosis. Importantly, VB‐RT NPs markedly attenuated these pathological changes, reducing interstitial edema and alveolar wall thickening, while oral PFD showed no obvious improvement. Masson's staining further confirmed extensive collagen deposition in the BLM group, which was substantially reduced by VB‐RT NPs treatment. Similarly, immunohistochemistry (IHC) staining demonstrated reduced expression of Collagen I and α‐SMA in VB‐RT NPs treated lungs (Figure 6B; Figure S29, Supporting Information). Additionally, body weight progressively declined in untreated fibrotic mice, while all treatment groups showed partial recovery. Notably, VB‐RT NPs‐treated mice regained body weight to levels comparable to those of the healthy control group (Figure 6C). Consistent with histological findings, untreated fibrotic mice exhibited a hydroxyproline (HYP) content 2.5‐fold higher than that of healthy donors. VB‐RT NPs treatment reduced HYP levels to 2.42‐fold, while PFD treatment achieved only a 1.70‐fold reduction relative to the BLM group (Figure 6D). As shown in Figure 6E, pathological scoring revealed severe fibrosis in the BLM group. All treatment groups showed decreased fibrosis scores, with the most pronounced improvement observed in the VB‐RT NPs group.

Integrins bind to RGD motifs present in latency‐associated peptides and proteins that bind TGF‐β, thereby promoting the activation of TGF‐β sequestered in the ECM.^[^ ^37^ ^]^ As shown in Figure 6B, IF results showed a significant positive correlation in TGF‐β1 LAP‐D (R58) staining intensity from the normal to the model group, with the lowest green fluorescence observed in the model group. This suggests that most TGF‐β1 LAP‐D in the model group is activated to active TGF‐β1 (Figure S30, Supporting Information). Interestingly, VB‐RT NPs treatment restored LAP‐D levels, suggesting a reduction in the release of active TGF‐β into the tissue microenvironment. Moreover, pro‐fibrotic cytokines TGF‐β and IL‐1β were significantly elevated in the BALF of the BLM group but markedly decreased following VB‐RT NPs treatment (Figure 6G,H). BALF VEGF was markedly elevated in the BLM group versus normal controls, consistent with injury‐/hypoxia‐driven secretion and increased vascular permeability. VB‐RT NPs reduced VEGF more effectively than single‐component controls, whereas PFD only partially decreased levels (Figure 6F). These data indicate that VB‐RT NPs attenuate pathological VEGF release rather than impairing angiogenic repair. Similar elevations of BALF VEGF after bleomycin injury and links to permeability have been reported previously, supporting our interpretation.^[^ ^38^, ^39^ ^]^ At the end of the treatment period, pulmonary function tests were performed to assess therapeutic efficacy across treatment groups. Parameters including tidal volume, minute ventilation, peak expiratory flow rate, and expiratory flow at 50% of lung volume were significantly improved, approaching values observed in healthy controls (Figure 6I–K; Figure S31, Supporting Information).

To assess the in vivo safety of the developed NPs, histological examination of the heart, liver, spleen, kidneys, and eyes revealed no histopathological abnormalities in NP‐treated groups (Figure S32, Supporting Information). Serum alanine aminotransferase (ALT) and aspartate aminotransferase (AST) levels showed no significant differences compared with healthy controls, indicating favorable biocompatibility. By contrast, mice receiving oral PFD exhibited significantly elevated ALT and AST levels, reflecting hepatotoxicity associated with systemic administration (Figure S32, Supporting Information). Taken together, these findings demonstrate that VB‐RT NPs not only provide superior anti‐fibrotic efficacy compared with oral PFD but also offer improved safety, likely due to targeted pulmonary delivery, enhanced local bioavailability, and reduced systemic toxicity.

### 2.7 VB‐RT NPs Modulate Mechanical Signaling and Improve the Mechanical Microenvironment in BLM‐Induced Fibrotic Mice

**Figure 7:**
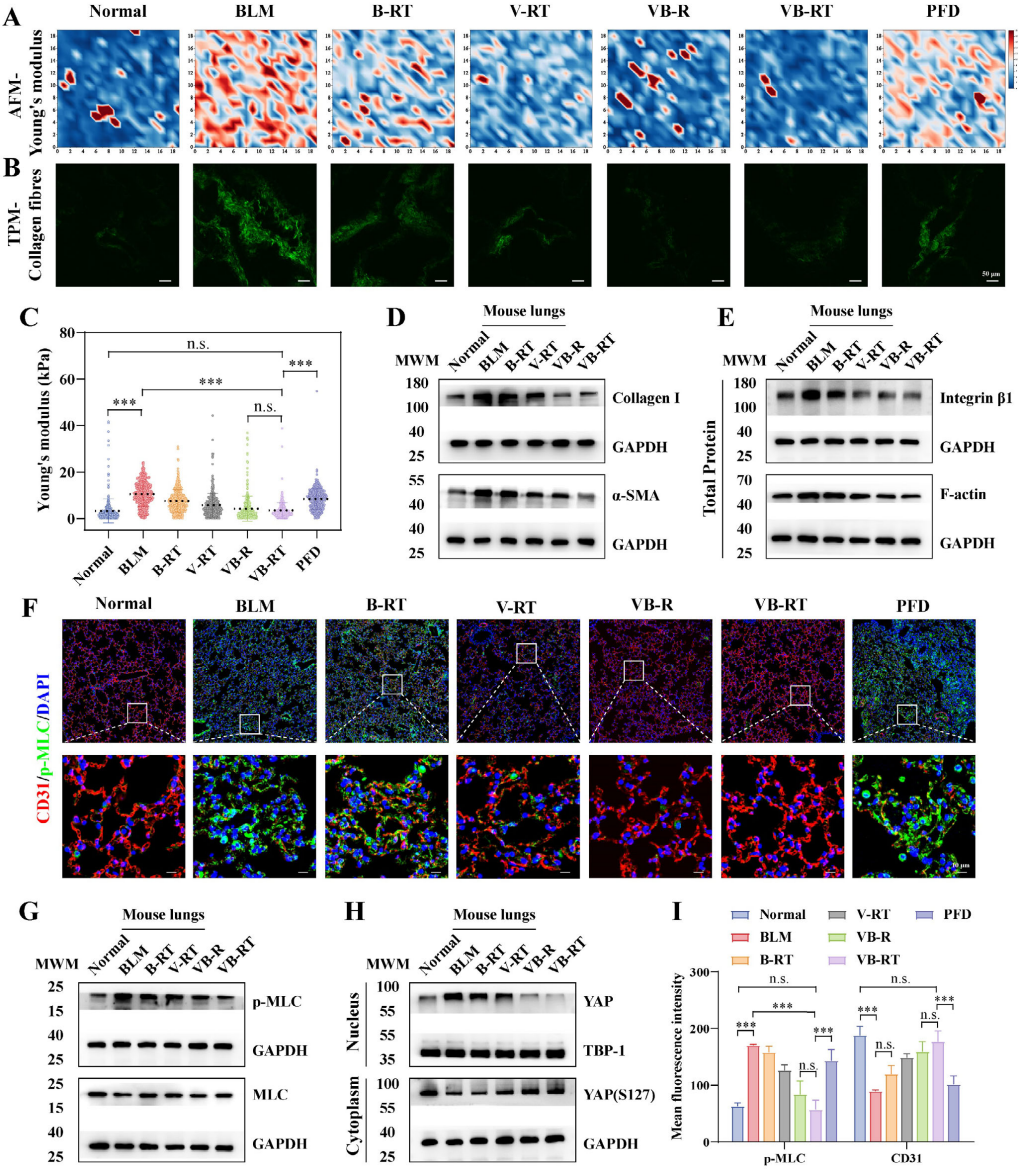
VB‐RT NPs modulate mechanical signaling and improve the mechanical microenvironment in BLM‐induced fibrotic mice. A) Representative heatmap of lung tissue stiffness in healthy, fibrotic, and treated mice, based on cantilever‐based AFM micro‐indentation analysis. B) Representative fluorescence images of collagen fibers in lung sections captured by two‐photon microscopy. C) Young's modulus of mice lungs was estimated by AFM indentation after different treatments (n = 200 measurements per lung tissue). D) Representative WB analysis of Collagen I and α‐SMA in lung tissues. E) Representative WB analysis of integrin β1 and F‐actin in lung tissues. F) Representative IF image of CD31 (red) and pMLC (green) in lung tissues. G) Representative WB analysis of pMLC and MLC in lung tissues. H) Representative WB analysis of YAP and YAP (S127) in cytoplasm and nucleus. I) Quantitative analysis of CD31 and pMLC fluorescence intensity using ImageJ (n = 3). All data are presented as the Mean ± SD. ^***^ *p* < 0.001. n.s., no significant difference.

To evaluate the impact of VB‐RT NPs on mechanical signaling and pulmonary biomechanics, the Young's modulus of lung tissue sections was measured and visualized using heatmaps. As shown in **Figure** **7**A, the model group exhibited a marked increase in overall stiffness compared to healthy controls, whereas all treatment groups showed varying degrees of stiffness reduction. VB‐RT NPs treatment reduced average pulmonary stiffness from 10.56 to 3.45 kPa, closely approximating that of healthy lungs (3.25 kPa). In comparison, B‐RT NPs and V‐RT NPs groups showed reductions to 7.56 and 5.71 kPa, respectively, while the VB‐R NPs group reached 4.16 kPa. Notably, lung stiffness in the oral PFD group remained elevated at 8.39 kPa, indicating limited efficacy in modulating the mechanical microenvironment (Figure 7C). Additionally, label‐free second‐harmonic generation imaging revealed pronounced collagen deposition in fibrotic lungs, further confirming ECM remodeling in IPF.^[^ ^40^ ^]^ The collagen fiber architecture observed in lung sections further supported the ability of VB‐RT NPs to modulate the fibrotic biomechanical microenvironment (Figure 7B). Consistently, WB analysis revealed that VB‐RT NPs significantly suppressed the upregulation of Collagen I and α‐SMA in fibrotic lungs (Figure 7D; Figure S33, Supporting Information).

Integrins are key mechanosensitive receptors that transduce ECM stiffness into intracellular biochemical cues, thereby activating Rho‐associated kinase and focal adhesion kinase, enhancing myosin II‐mediated contractility, and promoting nuclear translocation of YAP/TAZ to induce pro‐fibrotic gene expression.^[^ ^41^, ^42^ ^]^ To determine whether VB‐RT NPs modulate these processes, we examined integrin β1 and F‐actin expression. Both proteins were significantly upregulated in the BLM group, but their levels were progressively reduced in treatment groups, with the most substantial downregulation observed in VB‐RT NP‐treated lungs (Figure 7E; Figure S34, Supporting Information). Vascular remodeling was further assessed by CD31 staining to evaluate EC integrity. In fibrotic lungs, CD31 expression and capillary morphology were significantly disrupted, while VB‐RT NPs treatment restored pulmonary vascular structures and reduced fibrotic remodeling. Importantly, IF analysis showed elevated pMLC and reduced CD31 expression in fibrotic lungs, with pMLC levels in the PFD group remaining 1.2‐fold higher than in healthy controls. In contrast, VB‐RT NPs normalized pMLC expression and preserved the vascular niche, consistent with restored mechanical homeostasis (Figure 7F,I). These observations were corroborated by WB analysis of pMLC (Figure 7G; Figure S35, Supporting Information). In addition, nuclear YAP expression was significantly elevated in the BLM group, accompanied by decreased cytoplasmic YAP (S127), indicating enhanced nuclear translocation in fibrotic lungs. VB‐RT NPs treatment effectively suppressed this shift, thereby limiting activation of mechanosensitive signaling pathways (Figure 7H; Figure S36, Supporting Information). Together, these findings demonstrate that VB‐RT NPs remodel the fibrotic biomechanical microenvironment toward a more physiological state, restore mechanical homeostasis, and attenuate aberrant mechanotransduction, thereby inhibit IPF progression.

## 3 Discussions

IPF is a progressive and fatal interstitial lung disease in which aberrant signaling networks drive disease initiation and progression. Repeated alveolar epithelial injury provokes macrophage‐derived cytokines and growth factors, including TGF‐β and PDGF, which activate fibroblasts via SMAD and PI3K/AKT signaling to promote myofibroblast expansion, ECM deposition, and tissue remodeling.^[^ ^11^, ^43^ ^]^ In parallel, Ras activation in combination with TGF‐β has been shown to induce EndMT in ECs, further contributing to abnormal ECM production and fibrogenesis.^[^ ^44^ ^]^ Importantly, cellular responses during IPF are not confined to soluble mediators but are strongly influenced by mechanical cues. Progressive ECM accumulation increases tissue stiffness, which itself serves as a pathological stimulus.^[^ ^45^ ^]^ Fibroblasts sense this stiffened matrix through integrins and mechanosensors, activating YAP/TAZ to drive profibrotic gene transcription^[^ ^46^ ^]^ ECs also undergo mechanotransduction‐driven metabolic alterations that facilitate EndMT,^[^ ^20^ ^]^ while matrix stiffening upregulates TGF‐β and HIF‐1α expression and promotes the release of latent TGF‐β from the ECM.^[^ ^47^ ^]^ Moreover, mechanical tension promotes the release of latent TGF‐β from the ECM, amplifying biochemical signaling. Elevated mechanical stress further enhances TGF‐β signaling in alveolar type II epithelial cells, driving the centripetal progression of fibrosis from the lung periphery to the center.^[^ ^48^ ^]^ Taken together, biochemical signals increase cellular sensitivity to mechanical cues, while mechanical stress amplifies biochemical signaling, forming a self‐perpetuating feedback loop. Interrupting this pathological interplay may therefore offer a promising therapeutic strategy for IPF.

Our study demonstrates that simultaneous interruption of mechanical and biochemical signaling effectively suppresses their malignant crosstalk during fibrogenesis. The presence of TGF‐β stimulation markedly enhanced fibroblast contractility and activated mechanotransduction pathways, whereas VB‐RT NPs efficiently blocked this biochemical signal‐driven increase in mechanical force (Figure 3C–E). Conversely, under stiff matrix conditions, VB‐RT NPs significantly reduced the release of active TGF‐β and the nuclear translocation of pSMAD in fibroblasts, thereby attenuating biochemical signaling induced by mechanical cues (Figure 3F,G). Typically, when fibroblasts sense ECM stiffening, they further enhance ECM deposition and contraction, thereby establishing a self‐reinforcing positive feedback loop that accelerates fibrogenesis.^[^ ^49^ ^]^ Previous studies have confirmed that remodeling the fibrotic biomechanical microenvironment can disrupt this cycle. An inorganic ascorbic acid oxidase‐mimicking nanozyme loaded with liquiritigenin was shown to target collagen deposition and crosslinking, effectively reversing hepatic fibrosis in mice.^[^ ^42^ ^]^ Similarly, YAP and TAZ have been identified as key coordinators of matrix‐driven feedback loops, amplifying and sustaining pulmonary fibrosis.^[^ ^21^ ^]^ Consistent with these findings, our results demonstrated a marked reduction in YAP nuclear translocation following VB‐RT NPs treatment in fibrotic lungs (Figure 7H). In addition, previous studies reported that YAP can act as a transcriptional co‐regulator of SMAD3 to amplify TGF‐β signaling and drive pathological EndMT, highlighting YAP as a potential therapeutic target.^[^ ^50^ ^]^

In our study, mechanical forces promoted integrin β1 expression in ECs through integrin‐actin coupling, whereas VB‐RT NPs significantly improved endothelial tube formation and sprouting (Figure 4D,J). In addition, fibrotic stress induces EndMT, disrupts VE‐cadherin‐mediated junctions, and increases cellular contractility, whereas VB‐RT NPs effectively mitigated these pathological changes (Figure 4A,C). Mechanistically, increased matrix stiffness promotes integrin clustering and stabilizes focal adhesions, thereby sustaining cytoskeletal tension and facilitating organized vascular assembly. These results are in line with observations from a hyaluronic acid hydrogel model, which revealed that balanced matrix plasticity is essential for supporting endothelial growth and stable vascular formation, while excessive plasticity disrupts cell‐cell junctions.^[^ ^51^ ^]^ Together with our previous findings on the role of the pulmonary mechanical microenvironment in IPF, these results further substantiate the critical role of mechanical forces in initiating and sustaining the fibrotic cascade.^[^ ^52^ ^]^ More importantly, this combined therapeutic strategy targeting the evident synergy between mechanical and biochemical signaling significantly improved pulmonary function in vivo and remained effective even when administered at advanced stages of fibrosis, underscoring its applicability to chronic remodeling processes (Figure 6B). Compared with conventional strategies that selectively target either mechanical or biochemical signaling, VB‐RT NPs exhibited multifaceted antifibrotic effects by suppressing TGF‐β induced myofibroblast overactivation, markedly reducing fibroblast contractility, and promoting the restoration of endothelial vascular networks. Collectively, these integrated actions not only reversed fibrotic progression but also provided durable therapeutic benefits.

Patients with IPF often suffer from impaired respiratory function, a highly complex alveolar microenvironment, and dense collagenous ECM, all of which severely limit stable drug deposition and deep tissue penetration.^[^ ^53^ ^]^ To overcome these challenges, our multifunctional NPs were engineered with TA modification to scavenge ROS and inhibit lipid peroxidation.^[^ ^54^ ^]^ In addition, TA can bind to collagen structures, thereby enhancing selective accumulation in pathological regions.^[^ ^23^ ^]^ Meanwhile, L‐arginine modification promotes NO release from epithelial cells, induces MMP‐2/9 expression, and accelerates ECM degradation,^[^ ^55^ ^]^ thus improving tissue penetration (Figure 2N–P; Figure S13, Supporting Information). Further investigations confirmed that this formulation is capable of efficiently traversing the pulmonary mucus barrier (Figure S16, Supporting Information). Previous studies have likewise demonstrated that dual barrier‐penetrating inhaled liposome systems can overcome both mucus and ECM barriers, thereby enhancing pulmonary drug distribution and therapeutic efficacy.^[^ ^27^ ^]^ Our findings confirm that the integration of these features markedly improves drug delivery efficiency and highlights the translational potential of aerosolized nanomedicine for IPF. Importantly, the feasibility of clinical translation depends not only on efficacy but also on safety. Another noteworthy observation from our study is that the combined treatment did not cause any detectable structural or functional damage to the lungs or other major organs. Given that systemic administration of YAP inhibitors, as mechanotransduction regulators, can lead to long‐term safety concerns such as photosensitivity, hepatorenal toxicity, and impaired tissue regeneration, we specifically assessed serum biochemical parameters in mice and performed histological examinations of ocular tissues. Both results demonstrated that our formulation did not induce significant toxic effects (Figure S32, Supporting Information). This favorable safety profile is likely attributable to the pulmonary‐targeted delivery strategy, which enables precise drug accumulation at lesion sites while minimizing nonspecific injury to normal tissues. Although further studies are required to comprehensively evaluate long‐term safety and potential off‐target effects, our findings provide valuable evidence supporting the development of safer and more effective antifibrotic therapies. Using a lung‐targeted NP delivery system, we validated the feasibility of this approach in both in vitro and in vivo models, thereby demonstrating the therapeutic potential of an integrated antifibrotic strategy. Future work should focus on delineating broader networks of mechano‐biochemical signaling, exploring the feasibility of multi‐pathway combined inhibition, and extending this platform to other forms of fibrosis and therapy‐resistant pulmonary diseases. Collectively, this study establishes a dual‐targeting strategy against both mechanical and biochemical signaling, offering broad antifibrotic potential and providing a conceptual framework with significant translational relevance.

## 4 Conclusion

Herein, our study demonstrates that malignant between mechanical and biochemical signaling crosstalk exists in the lungs of IPF, and that this feedback loop can be disrupted by VB‐RT NPs, effectively halting the progression of IPF. In this approach, lipid NPs loaded with dual drugs were engineered and surface‐modified with L‐arginine and TA. These NPs not only target and eliminate excessive ROS in the IPF lungs, thereby localizing to the lung tissue, but also promote epithelial cells to release NO, facilitating NP penetration through the ECM into alveolar lesions. Moreover, the loaded BBM successfully suppresses the overactivation of fibroblasts and induces the reversion of myofibroblast‐like ECs to their endothelial state by inhibiting biochemical signal transduction. Simultaneously, VER inhibits the mechanosensitive effector YAP, blocking the activation of mechanical signals and thereby interrupting the malignant crosstalk between mechanical and biochemical signals. Following inhaled delivery, VB‐RT NPs were preferentially internalized by ECs and myofibroblasts, facilitating deep tissue penetration. In vivo, VB‐RT NPs significantly attenuated IPF, improved lung function, and restored the mechanical microenvironment in fibrotic mice. Collectively, our study highlights a promising therapeutic strategy to interrupt the malignant crosstalk between mechanical and biochemical signaling in IPF lungs. This strategy may also be applicable to other diseases involving similar signaling crosstalk and warrants further validation in diverse experimental models to evaluate its broader therapeutic potential.

## 5 Experimental Section

### Materials and Reagents

Phospholipid was obtained from AVT Pharmaceutical Tech Co., Ltd. (Shanghai, China), and Cholesterol from Sinopharm Chemical Reagent Co., Ltd. (Beijing, China). VER was purchased from Jiangsu Aikon Biopharmaceutical R&D Co., Ltd. (Nanjing, China), and PFD was purchased from Shanghai Accela ChemBio Co., Ltd. (Shanghai, China). L‐arginine was obtained from J&K Scientific Co., Ltd. (Beijing, China). BBM, DSPE‐PEG_2K_‐NH_2_, 1‐(3‐Dimethylaminopropyl)‐3‐ethylcarbodiimide hydrochloride (EDC·HCl), and N‐hydroxy succinimide (NHS) were purchased from Shanghai Aladdin Biochemical Technology Co., Ltd. (Shanghai, China). Mucin and TA were obtained from Shanghai Yuanye Bio‐Technology Co., Ltd. (Shanghai, China). Donkey serum was purchased from Solarbio Science & Technology Co., Ltd. (Beijing, China). Type I collagen (rat tail) was obtained from Corning (New York, USA). DAF‐FM DA and DCFH DA were obtained from Beyotime Biotechnology Co., Ltd. (Shanghai, China) and Sigma‐Aldrich, respectively. DiI and DiR were purchased from Shanghai BioScience Co., Ltd. (Shanghai, China). C6 was obtained from Tokyo Kasei Co., Ltd. (Tokyo, Japan). BLM was purchased from Hanhui Pharmaceuticals Co., Ltd. (Zhejiang, China). TGF‐β was purchased from PeproTech Inc. (NJ, USA). N‐Acetylcysteine (NAC) and N‐Nitro‐L‐arginine methyl ester (L‐NAME) were purchased from MedChemExpress (USA). FITC‐conjugated collagen was purchased from Abcam (UK). MMP‐2 and MMP‐9 assay kits were purchased from AiFang Biological (China). BCA reagent kit, Total protein extraction kit, TRIzol solution, Red Blood Cell Lysis Buffer, RPMI 1640 medium, and incomplete DMEM high sugar medium were purchased from Jiangsu Keygen Biotech Co., Ltd. (Jiangsu, China). The HYP detection kit was purchased from Nanjing Jiancheng Technology Co., Ltd. The Nuclear protein extraction kit was purchased from Shanghai BestBio Biotechnology Co., Ltd. The BlasTaq 2X qPCR Master Mix reagent kit was obtained from Applied Biological Materials Inc. Details of all antibodies used are listed in Table S1 (Supporting Information).

### Cell Culture

Primary lung fibroblasts were isolated from mice with IPF as described in the previous study.^[^ ^9^ ^]^ A549 cells were purchased from the National Collection of Authenticated Cell Cultures (Shanghai, China). PMVECs were purchased from ProCell, and 16HBE cells were obtained from ATCC (Shanghai, China). Primary lung fibroblasts were cultured in RPMI 1640 medium, while A549 cells, PMVECs, and 16HBE cells were maintained in high‐glucose DMEM.

### Human Samples and Animals

Human lung tissue samples were obtained from the Second Affiliated Hospital of Zhejiang University School of Medicine. Ethical review approval was obtained (No. I2023866). Male C57BL/6 mice (6–8 weeks) were purchased from Shanghai BK/KY Biotechnology Co., Ltd. All animal procedures were approved by the regional ethics committee of China Pharmaceutical University (Approval No. 2024‐10‐005) and conducted in accordance with institutional guidelines.

### Single‐Cell RNA Sequencing Data Analysis

Public single‐cell RNA‐seq data (GSE283885) were reanalyzed using Seurat (v5.0.1) in R (v4.3.1). Cells with 300–6000 detected genes, <20% mitochondrial transcripts, and log_10_(genes/UMI) > 0.8 were retained after quality control, and doublets were excluded with DoubletFinder. Data were normalized, highly variable genes identified, and principal component analysis (PCA) performed on the top 30 components, followed by batch correction using Harmony (v0.1.1). Clustering was conducted with the Louvain algorithm (resolution = 0.4) and visualized via UMAP. Cell types were annotated based on canonical markers (e.g., MS4A1, CD3D, COL1A1, CD163). Differentially expressed genes were identified by the Wilcoxon test (|log_2_FC| > 1, adjusted p < 0.05). GO and KEGG enrichment analyses were performed using clusterProfiler (v4.8.1) with org.Hs.eg.db and Benjamini‐Hochberg correction.

### Preparation and Characterization of NPs

VB NPs were prepared using the thin‐film dispersion method. Briefly, 40 mg of phospholipids, 5 mg of cholesterol, 5 mg of DSPE‐PEG_2K_‐NH_2_, 2 mg of VER, and 1 mg of BBM were dissolved in dichloromethane. The solvent was evaporated under reduced pressure using a rotary evaporator to form a homogeneous lipid film. The dried film was hydrated with PBS (5 mL) for 20 min and then sonicated in an ice bath for 5 min using a probe sonicator. Finally, the unmodified VB NPs were collected by centrifugation.

L‐arginine was dissolved in 5% glucose, then EDC and NHS were added for carboxyl activation at room temperature for 30 min. The pH was adjusted to 7.5. The activated L‐arginine was added to unmodified NPs and incubated overnight at room temperature. Catalyst and excess L‐arginine were removed by ultrafiltration to obtain L‐arginine modified VB‐R NPs. TA was dissolved in 5% glucose solution and adjusted to pH 7.5. VB‐R NPs were mixed with the TA solution and incubated at room temperature for 30 min. Free TA was removed by ultrafiltration to obtain TA‐functionalized VB‐RT NPs. Fluorescently labeled NPs were prepared similarly using C6, DiR, or DiI instead of drugs.

The particle size distribution and Zeta potential of the NPs were measured by Malvern Zetasizer Nano ZS. Morphology was observed by transmission electron microscopy (JEM‐200CX, JEOL, Japan). UV–vis spectra were recorded using a multifunctional microplate reader (ID5, Molecular Devices, China).

### Assessment of ROS Reduction

A549 cells were seeded in confocal culture dishes and co‐cultured with drug solutions (B‐RT, V‐RT, VB‐R, VB‐RT NPs) for 12 h. A positive control reagent for ROS was then added and incubated for 4 h. Subsequently, cells were kept in the dark and incubated with the fluorescent probe DCFH DA for 30 min. Finally, the images were captured.

### Evaluation of L‐arginine‐Induced NO Production

16HBE cells were seeded in a confocal dish and incubated with VB, VB‐R, and VB‐RT NPs for 4 h. After incubation, cells were washed with PBS and treated with the fluorescent probe DAF‐FM DA in the dark for 20 min. Nuclei were then stained with DAPI, and fluorescence images were acquired.

### Penetration of NPs through Pathological Barriers

16HBE cells were seeded in 35 mm^2^ culture dishes. An artificial mucus layer (0.3% w/v mucin solution) was applied and incubated overnight. C6‐labeled NPs were then added directly onto the mucus layer, and the cells were incubated in the dark for another 4 h. After incubation, the drug solution was discarded. The cell nuclei were stained with DAPI, and fluorescence images were acquired. Separately, 500 µL of type I collagen solution was added to the upper compartment of the Transwell chamber to form a collagen matrix, after which 16HBE cells were seeded. Myofibroblasts were pre‐seeded in the lower compartment. Subsequently, DiI‐labeled NPs were added to the chamber and incubated in the dark for 4 h. The drug solution in the chamber was then collected, and the levels of MMP‐2 and MMP‐9 were measured, while FITC‐conjugated collagen degradation was also observed. After processing the cells in the lower compartment, images were captured.

### Cell Uptake

Fibroblasts were pretreated with TGF‐β (10 ng mL^−1^) for 48 h to induce myofibroblast. Myofibroblasts were evenly seeded in the confocal culture dish, and incubated with Lipo/C6, Lipo/C6‐R, or Lipo/C6‐RT NPs for 4 h. PMVECs in 6‐well plates were treated with Lipo/DiI, Lipo/DiI‐R, or Lipo/DiI‐RT NPs for 4 h, harvested, and analyzed by flow cytometry (BD FACSCelesta, USA) to determine MFI. To assess the uptake of myofibroblasts under different ROS conditions, myofibroblasts were stimulated with L‐NAME (0.025 µM·ml^−1^·min^−1^) and NAC (1 mM) for 240 min. Subsequently, collagen penetration by the cells was observed, and images were captured.

### Release of Potential TGF‐β

Myofibroblasts were seeded into the chamber. After 3 days of culture, the cells were washed twice with pre‐cooled PBS. A lysis buffer containing 0.5% sodium deoxycholate, 1 mM phenylmethylsulfonyl fluoride (PMSF), and 10 mM Tris‐HCl (pH 8.0) was added and incubated at 4 °C for 10 min. After washing with PBS, a cleaning buffer containing 2 mM Tris‐HCl (pH 8.0) and 1 mM PMSF was added. Fibroblasts and PMVECs were then seeded onto the ECM layer in the chamber and cultured under tension or without tension. Various drug formulations (B‐RT, V‐RT, VB‐R, VB‐RT NPs) were added for stimulation. After treatment, cells in the chamber were collected, centrifuged to obtain the supernatant, and the TGF‐β content was measured.

### 3D Collagen Gel Contraction Assay

The pH of type I collagen was adjusted using NaOH (0.1 M). A total of 200 µL of type I collagen and 800 µL of cell suspension (1 × 10⁵ cells) were mixed and added to a 24‐well plate, then incubated at 37 °C with 5% CO_2_ for 1 h. After treatment, gel images were captured at 48 h, and the gel area was quantified using ImageJ software (NIH, MD, USA).

### Live Cell Imaging Experiment

A total of 500 µL of PMVEC suspension (5 × 10⁴ cells) and 500 µL of fibroblast suspension (5 × 10⁴ cells) were added to a 29 mm^2^ glass‐bottomed culture dish and incubated at 37 °C with 5% CO_2_ for 1 h. CellMask actin tracer and CellTracker Red CMTPX dye were then added, followed by incubation for 30 min. To reduce background fluorescence and maintain stable pH during imaging, the culture medium was discarded and replaced with EBSS buffer. Finally, the dishes were placed in a live‐cell imaging workstation (Delta Vision Ultra, GE, USA), and images were acquired at a frame rate of 10 min for 70 min.

### Programmable Mechanical Cell Stretch System

Fibroblasts were seeded into the small chambers of a programmable cell stretch and tension system (Beatle, Cell & Force, China). After 24 h, various drug treatments were applied. Fibroblasts were then subjected to cyclic mechanical stretching (Mode: SIN F; amplitude: 5; duration: 20) for 24 h. Subsequently, α‐SMA expression was evaluated by IF staining, and images were acquired using a confocal laser scanning microscope (CLSM‐800, Zeiss, Germany).

### EC Tube Formation Experiment

A total of 50 µL of matrix gel solution at varying concentrations was added to a 96‐well plate to form gels with different stiffness. Then, 100 µL of PMVEC suspension (1 × 10⁴ cells) was added to each well. When cell confluence reached ≈80%, the culture medium was removed, and various drug treatments were applied. Images were captured at 6 h and 12 h post‐treatment, and the number of branching points and tube lengths were quantified using ImageJ software (NIH, MD, USA).

### Preparation of Soft and Stiff Gels

Collagen protein was diluted with DPBS to final concentrations of 0.5–1 mg mL^−1^ (soft) and 3.0–3.5 mg mL^−1^ (stiff). The pH was adjusted to 7.4 using 1 M NaOH. A total of 125 µL of the mixture was added to a 35 mm^2^ Petri dish and polymerized for 60 min at 37 °C in 5% CO_2_ to form a gel layer with a thickness of 60–70 µm.

### Staining of 3D Soft and Stiff Gels

Add 1 × 10^6^ PMVECs to the collagen solution such that the PMVEC suspension volume is one‐eighth of the collagen solution volume. Adjust the pH of the mixture to 7.4. Then, dispense 200 µL of the collagen‐EC mixture into a non‐TC‐treated 48 well plate and incubate at 37 °C with 5% CO_2_ for 60 min to allow gelation. Next, add 400 µL of incomplete medium, and use a pipette to draw a line around the gel to detach it from the well wall and allow it to float. After culturing for 48 h, remove the medium and apply the desired stimuli. Finally, IF staining of pMLC and F‐actin was performed, and fluorescence images were acquired.

### Young's Modulus Measurement

Human and mouse lung tissues were sectioned into 100 µm‐thick slices using a vibrating microtome (Leica VT1000S, China). The slices were then mounted onto culture dishes and immersed in pre‐cooled PBS. The Young's modulus of human lung tissue was measured using a nanoindentation system (Pavone, Optics 11 Life, Netherlands). A 5 × 5 single‐point indentation grid was applied using a spherical probe with a stiffness of 0.017 N m^−1^ and a radius of 3 µm. Mechanical force‐displacement curves were recorded and fitted using the Hertz model to calculate the Young's modulus. Topographical heat maps and modulus values were output accordingly. AFM was performed in force mapping mode to assess the mechanical properties of mouse lung tissue using an AFM system (NT‐AIST, HORIBA, Japan) equipped with high‐quality probes (MikroMasch, USA). The spring constant of the cantilever was 0.5 N m^−1^. Topographical maps and Young's modulus distributions were obtained to characterize the biological structure. To measure the modulus of both cells and lung tissue, force‐indentation mapping was conducted over a 20 µm × 20 µm scanning area using a 20 × 20 grid of force curves.

### Traction Force Detection

The TFM slide was observed under an inverted microscope. The focal plane was adjusted to the top of the microcolumn, followed by fine‐tuning to focus on the bottom surface. Images of the microcolumns were captured before cell seeding for reference. Then, 2 mL of culture medium was added to a glass‐bottom Petri dish, and 200 µL of fibroblast suspension (1 × 10⁵ cells) was gently added onto the microcolumn array to establish fibroblast cultures. After 24 h of incubation, the culture medium was removed and replaced with 2 mL of incomplete medium. Cells were cultured for another 10 h to allow return to the resting state. The slides were then re‐observed under the inverted microscope. Cell deformation and displacement at the top of the PDMS microcolumns were monitored in real time to assess the traction force upon different formulations stimulation.

### Western Blot Assay

First, proteins were extracted from cells or lung tissues, and their concentrations were determined using a commercial kit. After separation by electrophoresis, the proteins were transferred onto a nitrocellulose membrane. The membranes were then blocked with 5% skimmed milk powder and incubated with primary antibodies at 4 °C overnight, followed by incubation with secondary antibodies at room temperature for 2 h. Protein bands were visualized using a CCD imaging system (Tanon 4200, Shanghai, China), and densitometric analysis of target protein expression was performed using ImageJ software (NIH, MD, USA).

### Establishment of the IPF Model

After one week of adaptive feeding of C57 mice, a single intratracheal injection of 2.0 U kg^−1^ BLM was administered. On the 14th day, an experimental pulmonary fibrosis model was established.

### Evaluation of Therapeutic Effects of NPs in the Mice Model

On days 14, 16, 18, 20, 22, and 24 post‐modeling, mice received aerosol inhalation of normal saline, B‐RT, V‐RT, VB‐R, and VB‐RT NPs (VER: 10 mg kg^−1^, BBM: 8 mg kg^−1^). An additional group received oral gavage of PFD (25 mg kg^−1^). Body weight changes were monitored throughout the treatment period. The nebulization system used for drug delivery was purchased from Yuyan Instruments (Shanghai, China).

### Biodistribution of NPs In Vivo

After establishing a BLM‐induced pulmonary fibrosis model in C57 mice, DiR/DiI‐labeled NPs were administered via aerosol inhalation. At 24 h post‐inhalation, mice were imaged using an IVIS system (Tanon, China) to visualize NP distribution in the lungs. Subsequently, major organs‐including the heart, liver, spleen, lungs, and kidneys‐were harvested to assess NP distribution. To further evaluate NP retention in the lungs, dissected lung tissues were immediately placed on dry ice, fixed, and sectioned for IF staining (CD31, FAP). Fluorescence intensity was quantified across different groups.

### Distribution of NPs in BALF

DiI‐labeled NPs were administered to each group via nebulization following model establishment. The BALF of each group of mice was collected at 1, 12, and 24 h, respectively, and centrifuged. The MFI of DiI in both the supernatant and cell pellet was measured using a multifunctional microplate reader (ID5, Molecular Devices, China) and a flow cytometer (BD FACSCelesta, USA), respectively.

### Detection of Cytokines in BALF

After six doses of treatment were administered to each group of mice, BALF was collected. The concentrations of TGF‐β, IL‐1β, and VEGF in the BALF were quantified using ELISA kits.

### HYP and Histological Analysis

HYP levels in lung tissue were measured using a HYP assay kit. Major organs were harvested, and paraffin‐embedded tissue sections were prepared for H&E, Masson's trichrome, and IHC staining.

### Pulmonary Function Test

Whole‐body plethysmography (DSI Buxco, WBP, USA) was used to assess respiratory parameters in mice. Animals were placed in a volume chamber to maintain stable breathing, with four mice tested per session. Each mouse was identified and recorded via the emission station. Measured respiratory parameters included tidal volume, minute ventilation, peak expiratory flow rate, and expiratory flow rate at 50%.

### Quantitative RT‐PCR

Total RNA was extracted from ground lung tissue using TRIzol solution, and cDNA was synthesized using the BlasTaq 2X qPCR Master Mix kit. mRNA expression levels were quantified by the 2^−ΔΔCT^ method using GAPDH as an internal reference. Primer sequences are listed in Table S2 (Supporting Information).

### Statistical Analysis

All data were analyzed using GraphPad Prism 8.0.1 software. Data were checked for normal distribution and outliers before analysis and are presented as mean ± SD (n ≥ 3). Statistical significance was determined using one‐way ANOVA followed by Tukey's multiple comparison test or a two‐tailed Student's t‐test, with significance levels defined as p < 0.05 (ns: p > 0.05, ^*^ *p* < 0.05, ^**^ *p* < 0.01, and ^***^ *p* < 0.001). For experiments involving two variables, two‐way ANOVA was also performed to assess interaction effects.

## Conflict of Interest

The authors declare no conflict of interest.

## Supporting information



Supporting Information

## Data Availability

The data that support the findings of this study are available from the corresponding author upon reasonable request.
