# Supplementary material for: Pulmonary‐Targeted Nanoparticles Interrupt the Malignant Mechanical and Biochemical Signaling Crosstalk for Idiopathic Pulmonary Fibrosis Therapy
Source: Adv Sci (Weinh). 2025 Nov 17;13(5):e12658. doi: 10.1002/advs.202512658 (PMC12850245; doi:10.1002/advs.202512658)
Supplement: Supplementary file 1 — Supporting Information [file ADVS-13-e12658-s001.pdf]

Supporting Information

Pulmonary-Targeted Nanoparticles Interrupt the Malignant Mechanical and Biochemical Signaling Crosstalk for Idiopathic Pulmonary Fibrosis Therapy

Xue-Na Li [a, b †], *Ya-Ping Lin* [c, †], *Xi-Xi Ma* [c], *Yue-Fei Fang* [c], *Hui Wang* [f], *Chun-Hui Cui* [c], *Chen Zhang* [c], *Jin-You Piao* [b], *Jee-Heon Jeong* [e], *Xian-Wu Cheng* [a, *], *Lei Xing* [c, *], *Hu-Lin Jiang* [a, b, c, d, e *]

[a] Department of Cardiology and Hypertension, Affiliated Hospital of Yanbian University, Yanji 133000, China.

[b] College of Pharmacy, Yanbian University, Yanji 133000, China.

[c] State Key Laboratory of Natural Medicines, Department of Pharmaceutics, China Pharmaceutical University, Nanjing 210009, China.

[d] Joint International Research Laboratory of Target Discovery and New Drug Innovation, MOE, China Pharmaceutical University, Nanjing 210009, China.

[e] Department of Precision Medicine, School of Medicine, Sungkyunkwan University, Suwon 16419, South Korea.

[f] Department of Endocrinology, Zhongda Hospital, School of Medicine, Southeast University, Nanjing 210009, China.

[*]Corresponding Author(s). E-mail(s): jianghulin3@163.com or jianghulin3@cpu.edu.cn; xinglei6xl@163.com; chengxw@ybu.edu.cn.

[†]These authors contributed equally to this work.

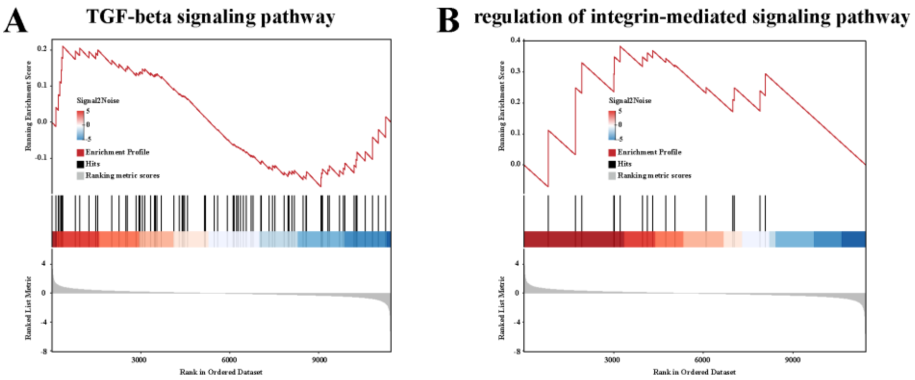

Figure S1. GSEA of signaling pathways in ECs. A) GSEA analysis showed the gene sets of TGF-β signaling pathway in ECs. B) GSEA analysis showed the gene sets of regulation of integrin-mediated signaling pathway in ECs.

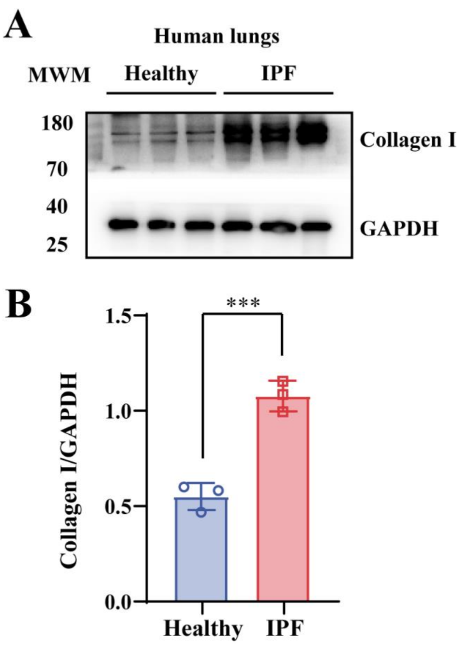

Figure S2. WB analysis in healthy donors and IPF patient lungs. A) Representative WB assays of Collagen I in healthy donors and IPF patient lungs. B) The Collagen I/GAPDH ratio in human samples was analyzed using ImageJ (n = 3). All data are presented as the Mean ± SD. ***$p < 0.001$.

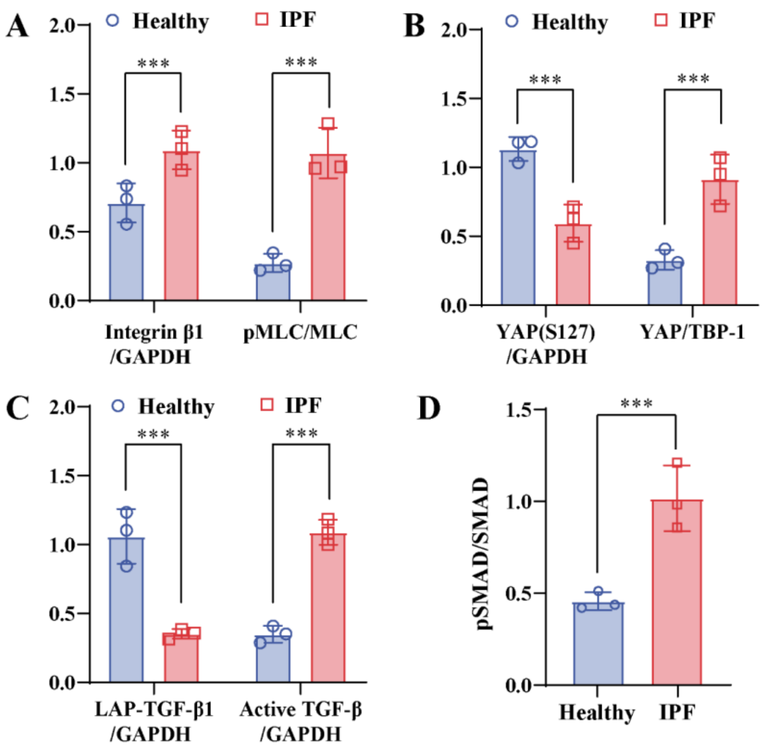

Figure S3. Quantification of protein expression in lung tissues from healthy donors and IPF patients. A) Protein levels of integrin β1 and pMLC normalized to GAPDH and total MLC, respectively (n = 3). B) Relative expression of cytoplasmic YAP (S127)/GAPDH and nuclear YAP/TBP-1 (n = 3). C) Protein levels of LAP-TGF-β1 and active TGF-β normalized to GAPDH (n = 3). D) Ratio of pSMAD to total SMAD (n = 3). All data are presented as the Mean ± SD. ***$p < 0.001$.

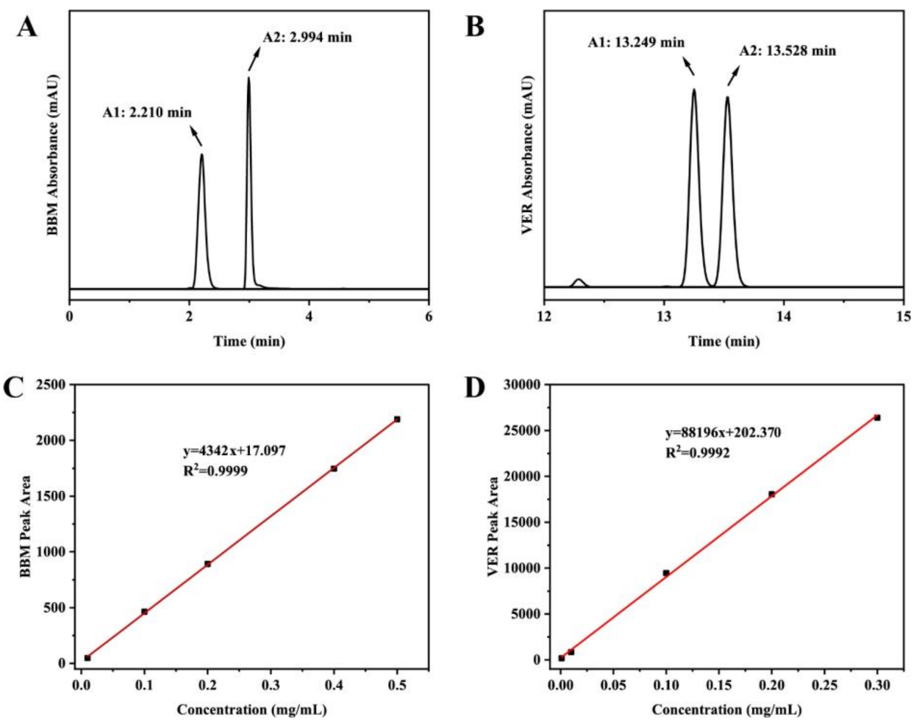

Figure S4. HPLC analysis and standard calibration curves of BBM and VER. A) HPLC chromatogram of BBM with retention times at 2.210 min and 2.994 min. B) HPLC chromatogram of VER with retention times at 13.249 min and 13.528 min. C) Standard calibration curve of BBM showing the relationship between concentration and peak area. D) Standard calibration curve of VER showing the relationship between concentration and peak area.

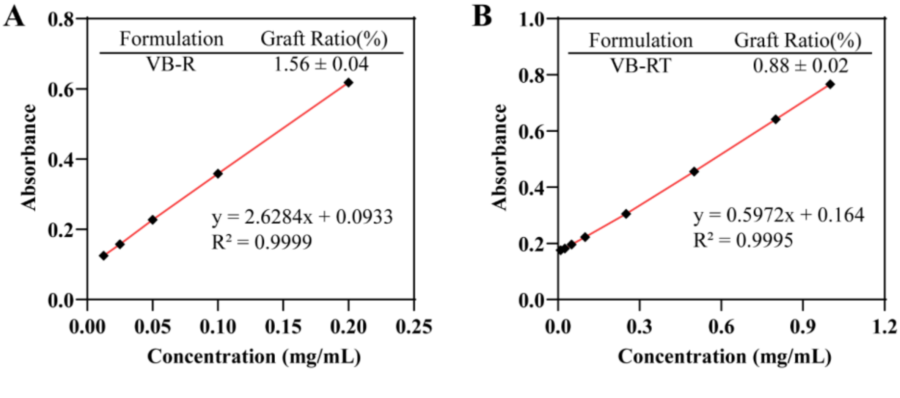

Figure S5. Standard calibration curves and grafting ratio quantification of L-arginine and TA. A) Calibration curve of L-arginine for absorbance versus concentration, with a measured grafting ratio of 1.56 ± 0.04%. B) Calibration curve of TA for absorbance versus concentration, with a measured grafting ratio of 0.88 ± 0.02%.

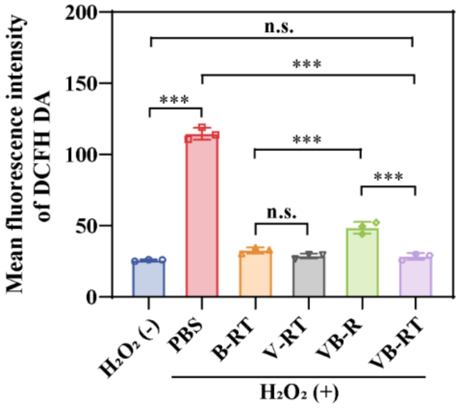

Figure S6. Quantification of ROS-associated MFI in 16HBE cells labeled with DCFH DA under different treatments (n = 3). All data are presented as the Mean ± SD. ***p < 0.001. n.s., no significant difference.

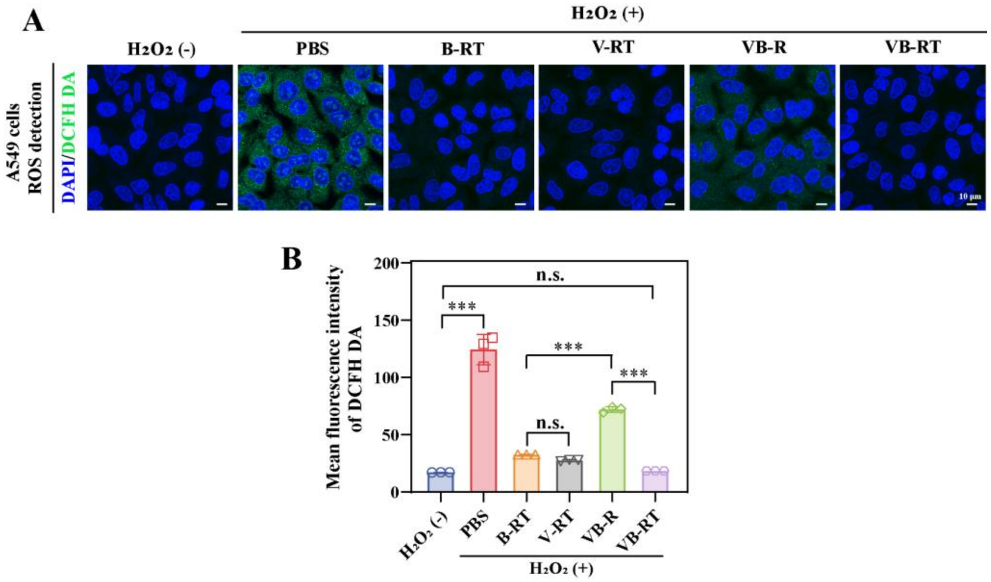

Figure S7. ROS detection and quantification in A549 cells under different treatments. A) Fluorescence imaging of ROS levels in A549 cells under different treatments using DCFH DA. B) Quantification of ROS-associated MFI in A549 cells labeled with DCFH DA (n = 3). All data are presented as the Mean ± SD. ***p < 0.001. n.s., no significant difference.

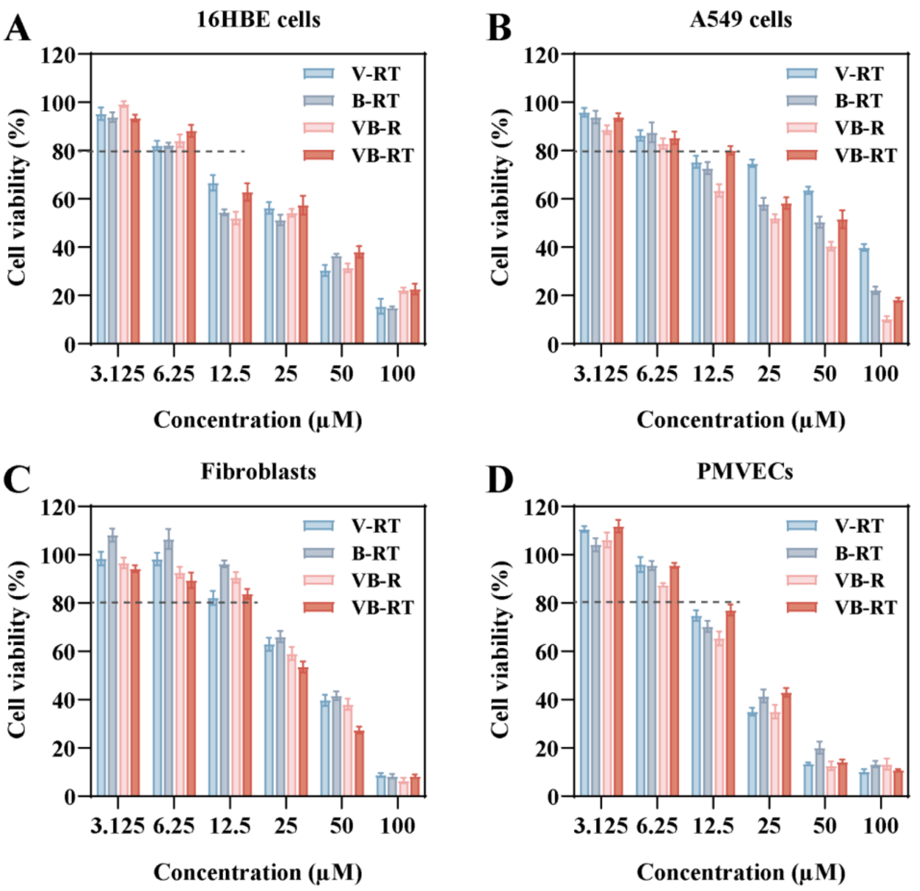

Figure S8. *In vitro* cytotoxicity of different formulations in 16HBE cells (A), A549 cells (B), fibroblasts (C), and PMVECs (D) (n = 6).

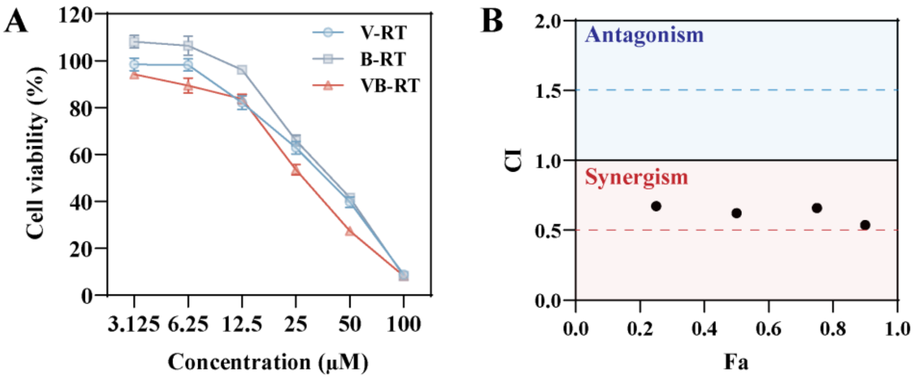

Figure S9. Evaluation of fibroblast viability and synergistic effects of V-RT NPs, B-RT NPs, and VB-RT NPs at different concentrations. A) Cell viability of V-RT NPs, B-RT NPs, and VB-RT NPs at varying concentrations (n = 6). B) Fa-CI curve showing synergism (CI < 1) for all combinations across varying effect levels.

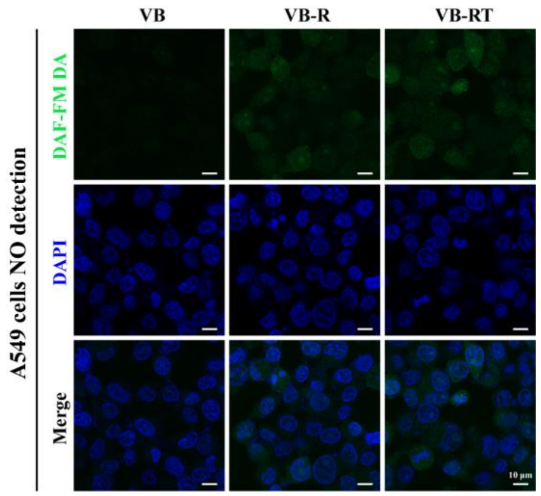

Figure S10. The NO detection in A549 cells under different treatments using DAF-FM DA.

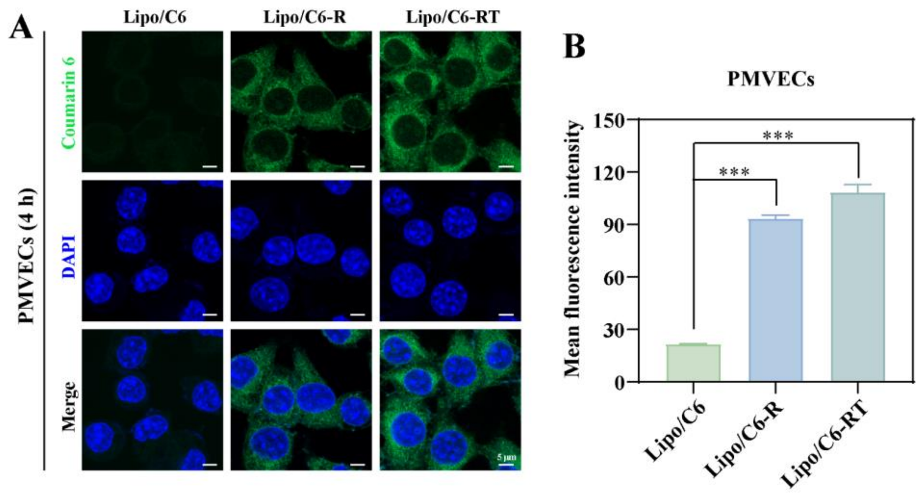

Figure S11. Cellular uptake of different formulations in PMVECs. A) CLSM images of PMVECs treated with different formulations for 4 h at 37 °C. B) Quantification of fluorescence intensity in PMVECs analyzed using ImageJ (n = 3). All data are presented as the Mean ± SD. ***$p < 0.001$.

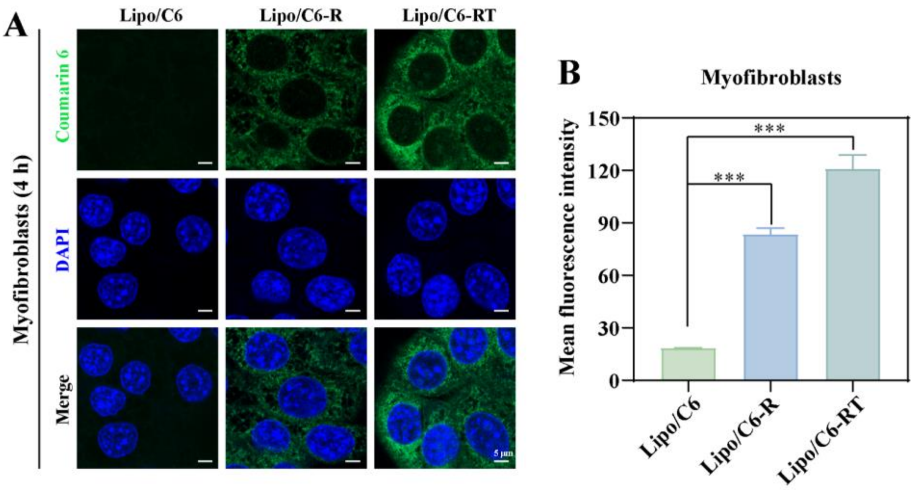

Figure S12. Cellular uptake of different formulations by myofibroblasts. A) CLSM images of myofibroblasts treated with different formulations for 4 h at 37 °C. B) Quantification of fluorescence intensity in myofibroblasts analyzed using ImageJ (n = 3). All data are presented as the Mean ± SD. ***p < 0.001.

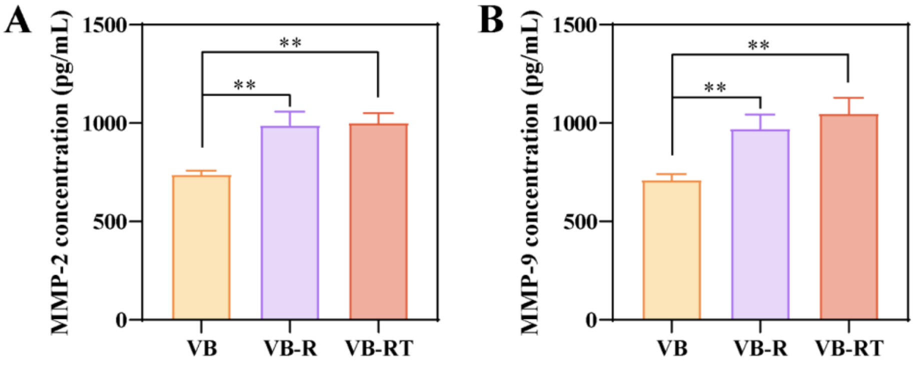

Figure S13. MMP-2 and MMP-9 secretion by epithelial cells after exposure to different NP formulations. (A) Quantification of MMP-2 levels in the conditioned media of epithelial cells treated with VB NPs, VB-R NPs, or VB-RT NPs (n = 3). (B) Quantification of MMP-9 levels under the same treatment conditions (n = 3).

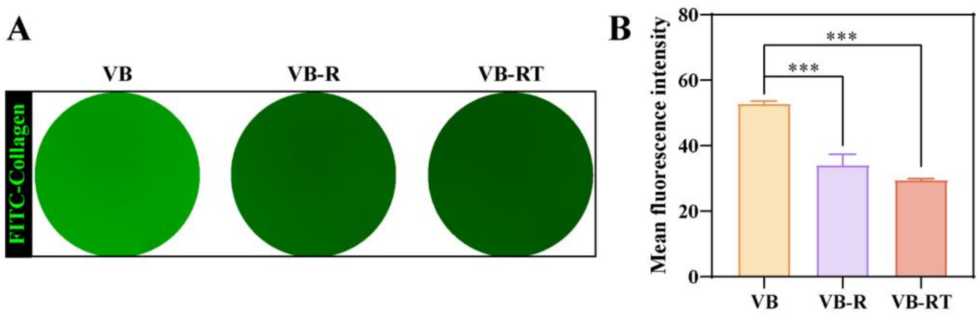

Figure S14. Collagen quantification after treatment with different formulations. (A) Representative FITC-collagen images of fibroblasts treated with VB NPs, VB-R NPs, and VB-RT NPs. (B) Quantification of collagen levels based on FITC-collagen fluorescence intensity (n = 3). All data are presented as the Mean ± SD. ***p < 0.001.

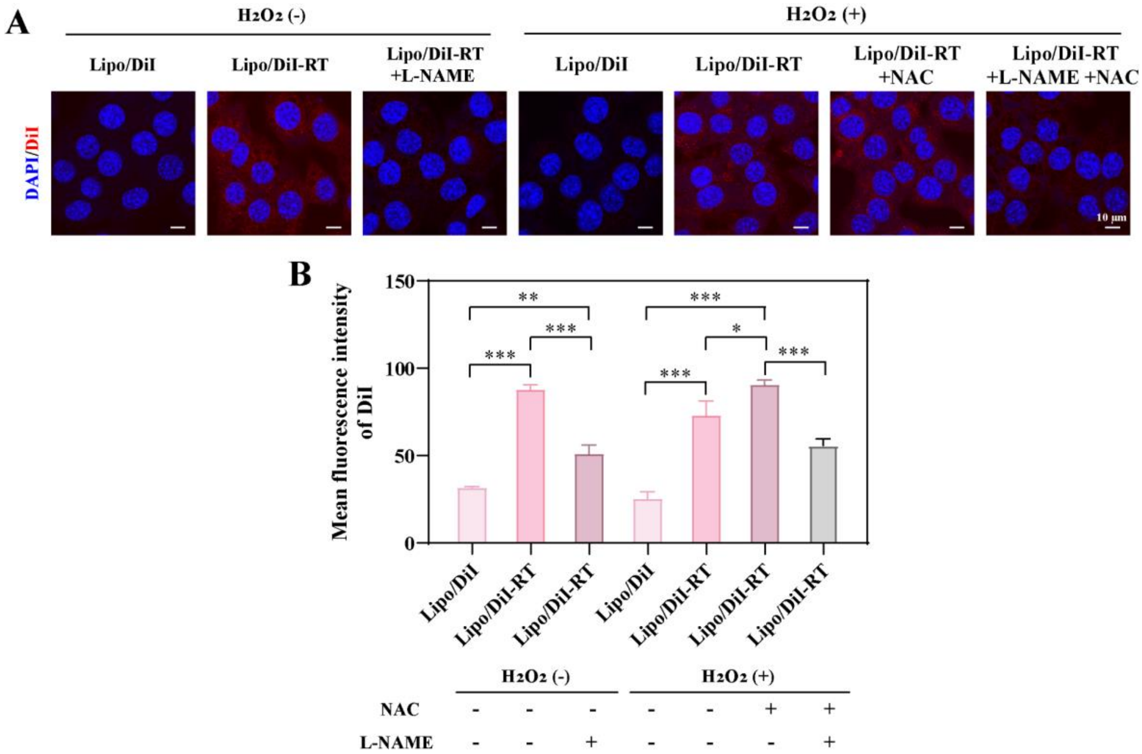

Figure S15. Cellular uptake of DiI-labeled liposomes (Lipo/DiI and Lipo/DiI-RT) by fibroblasts in a Transwell co-culture system. (A) Representative fluorescence images showing DiI (red) and nuclei (DAPI, blue) in fibroblasts cultured in the lower chamber, following treatment of the upper 16HBE cells with or without H_2O_2, L-NAME, or NAC. (B) Quantification of mean DiI fluorescence intensity in fibroblasts (n = 3). All data are presented as the Mean ± SD. *p < 0.05, **p < 0.01, and ***p < 0.001.

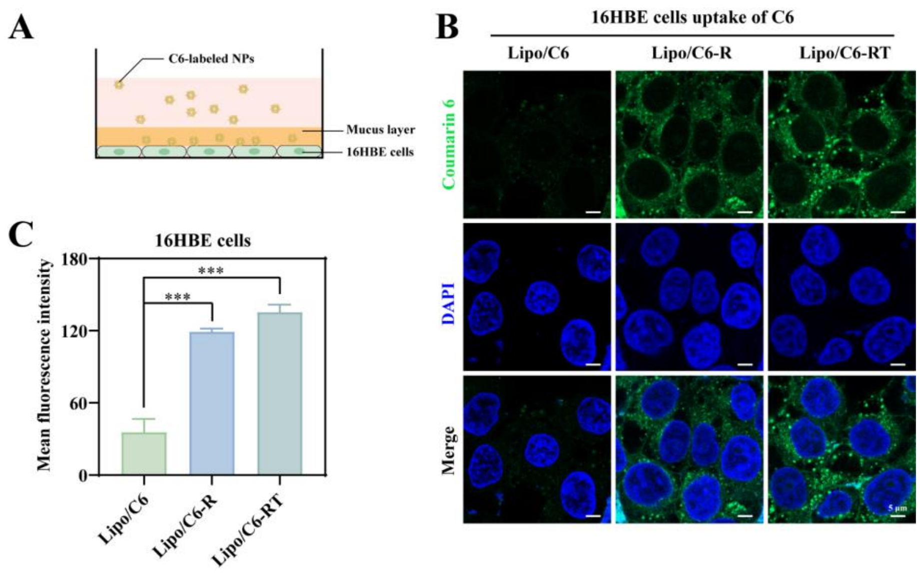

Figure S16. Cell uptake of different formulations in 16HBE cells with mucus layer. A) Schematic of C6-labeled NPs uptake by 16HBE cells with a mucus layer. B) Confocal images showing the uptake of different formulations. C) Quantification of fluorescence intensity in 16HBE cells analyzed using ImageJ (n = 3). All data are presented as the Mean ± SD. ***$p < 0.001$.

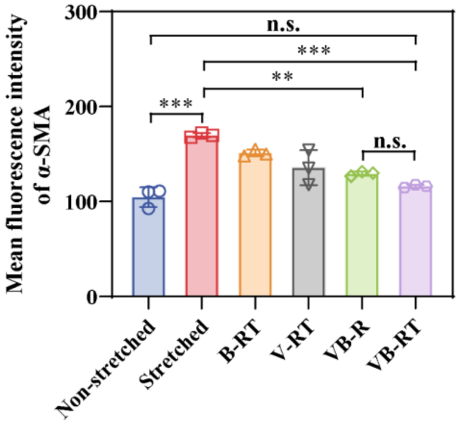

Figure S17. Quantification of MFI of a-SMA in fibroblasts using ImageJ (n = 3). All data are presented as the Mean ± SD. **$p < 0.01$, and ***$p < 0.001$. n.s., no significant difference.

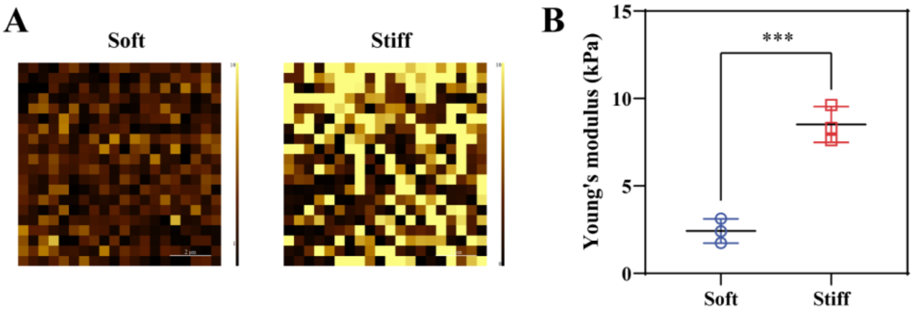

Figure S18. Young's modulus of soft and stiff matrices. (A) Representative AFM elastic modulus maps of soft and stiff matrices. (B) Quantification of Young's modulus for soft and stiff matrices (n = 3). All data are presented as the Mean ± SD. ***p < 0.001.

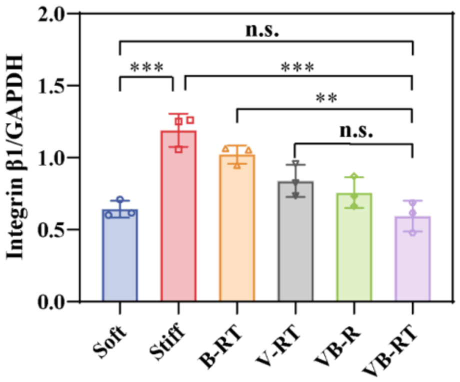

Figure S19. Quantitative analysis of integrin β1 expression in fibroblasts based on the integrin β1/GAPDH ratio determined using ImageJ. All data are presented as the Mean ± SD. **p < 0.01, and ***p < 0.001. n.s., no significant difference.

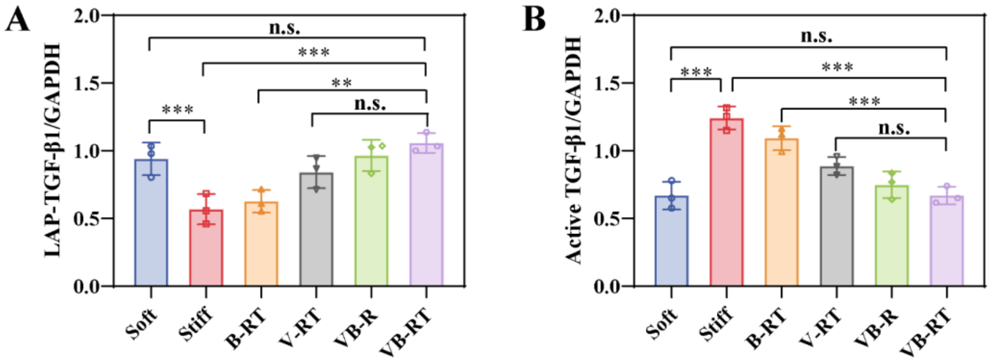

Figure S20. The LAP-TGF-β1/GAPDH ratio (A) and active TGF-β1/GAPDH ratio (B) in fibroblasts analyzed using ImageJ (n = 3). All data are presented as the Mean ± SD. **p < 0.01,

and ***p < 0.001. n.s., no significant difference.

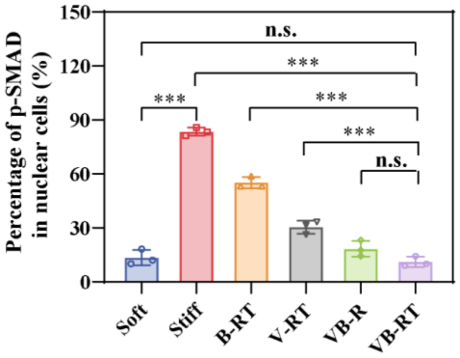

Figure S21. Percentage of nuclear p-SMAD-positive cells in different groups analyzed (n = 3). All data are presented as the Mean ± SD. ***p < 0.001. n.s., no significant difference.

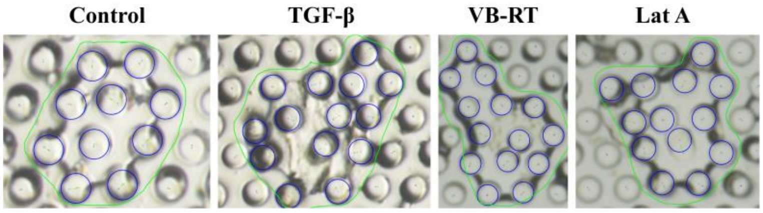

Figure S22. Traction force image of fibroblasts on micropillar substrates under different conditions.

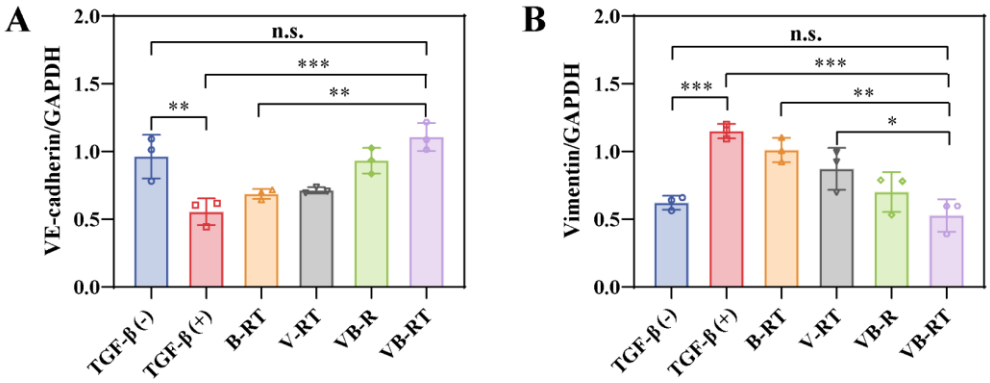

Figure S23. The VE-cadherin/GAPDH ratio (A) and Vimentin/GAPDH ratio (B) in PMVECs analyzed using ImageJ (n = 3). All data are presented as the Mean ± SD (n = 3). *p < 0.05, **p < 0.01, and ***p < 0.001. n.s., no significant difference.

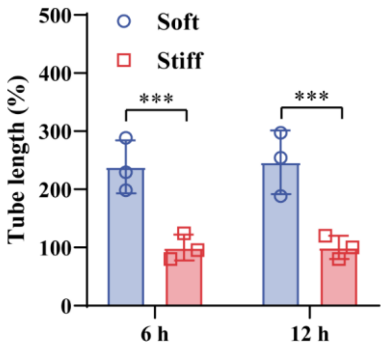

Figure S24. Quantification of tube length in tube formation assay on soft and stiff matrices at 6 h and 12 h (n = 3). All data are presented as the Mean ± SD. ***p < 0.001.

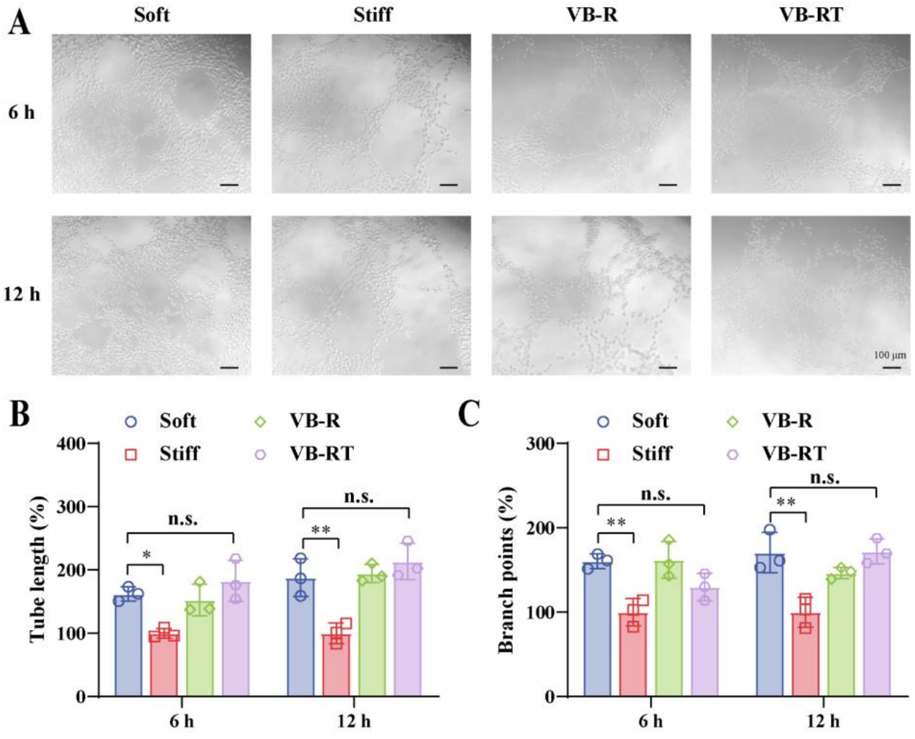

Figure S25. VB-RT NPs enhance angiogenic capacity of PMVECs on matrices with varying stiffness. A) Tube formation on soft and stiff matrices after treatment at 6 h and 12 h. B) Quantification of tube length in tube formation on soft and stiff matrices after treatment at 6 h and 12 h (n = 3). C) Quantification of branch points in tube formation assay on soft and stiff matrices after treatment at 6 h and 12 h (n = 3). All data are presented as the Mean ± SD. *p < 0.05, **p < 0.01, and n.s., no significant difference.

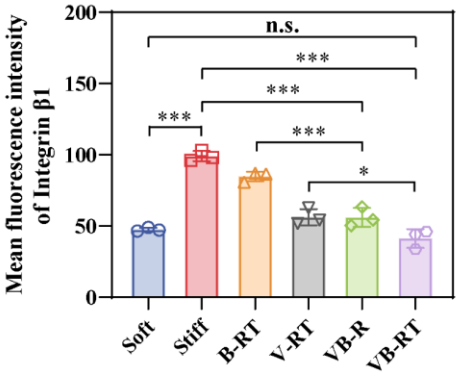

Figure S26. Quantification of MFI of integrin β1 in PMVECs using ImageJ (n = 3). All data are presented as the Mean ± SD. *p < 0.05, ***p < 0.001. n.s., no significant difference.

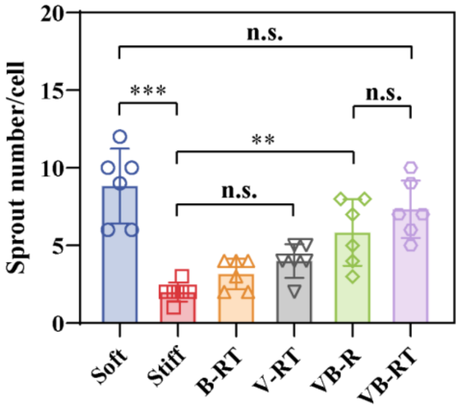

Figure S27. Quantification of sprout number per cell from encapsulated PMVEC spheroids in soft and stiff matrices at 24 h (n = 6). All data are presented as the Mean ± SD. **p < 0.01, and ***p < 0.001. n.s., no significant difference.

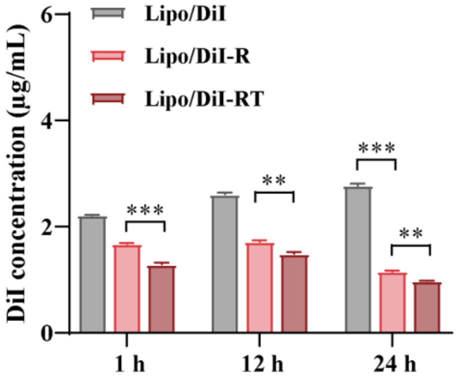

Figure S28. DiI concentration in the supernatant of BALF (n = 3). All data are presented as the

Mean ± SD. **p < 0.01, and ***p < 0.001.

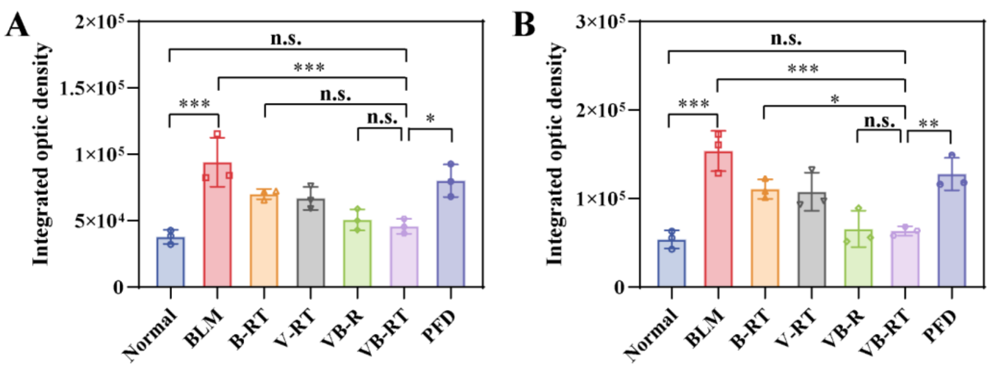

Figure S29. Quantification of Collagen I (A) and α-SMA (B) IHC staining in mouse lung tissue using ImageJ (n = 3). All data are presented as the Mean ± SD (n = 3). *p < 0.05, **p < 0.01, and ***p < 0.001. n.s., no significant difference.

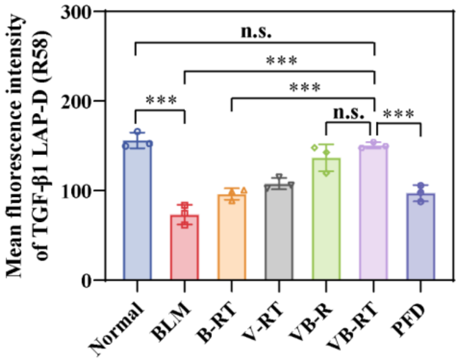

Figure S30. Quantification of MFI of TGF-β1 LAP-D (R58) in mouse lung tissue using ImageJ (n = 3). All data are presented as the Mean ± SD (n = 3). ***p < 0.001. n.s., no significant difference.

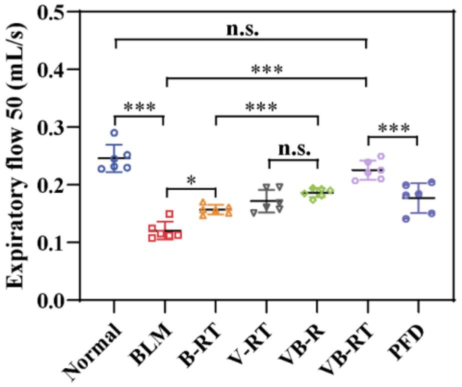

Figure S31. Lung function analysis of expiratory flow at 50% in each group (n = 6). All data are presented as the Mean ± SD. *p < 0.05, ***p < 0.001. n.s., no significant difference.

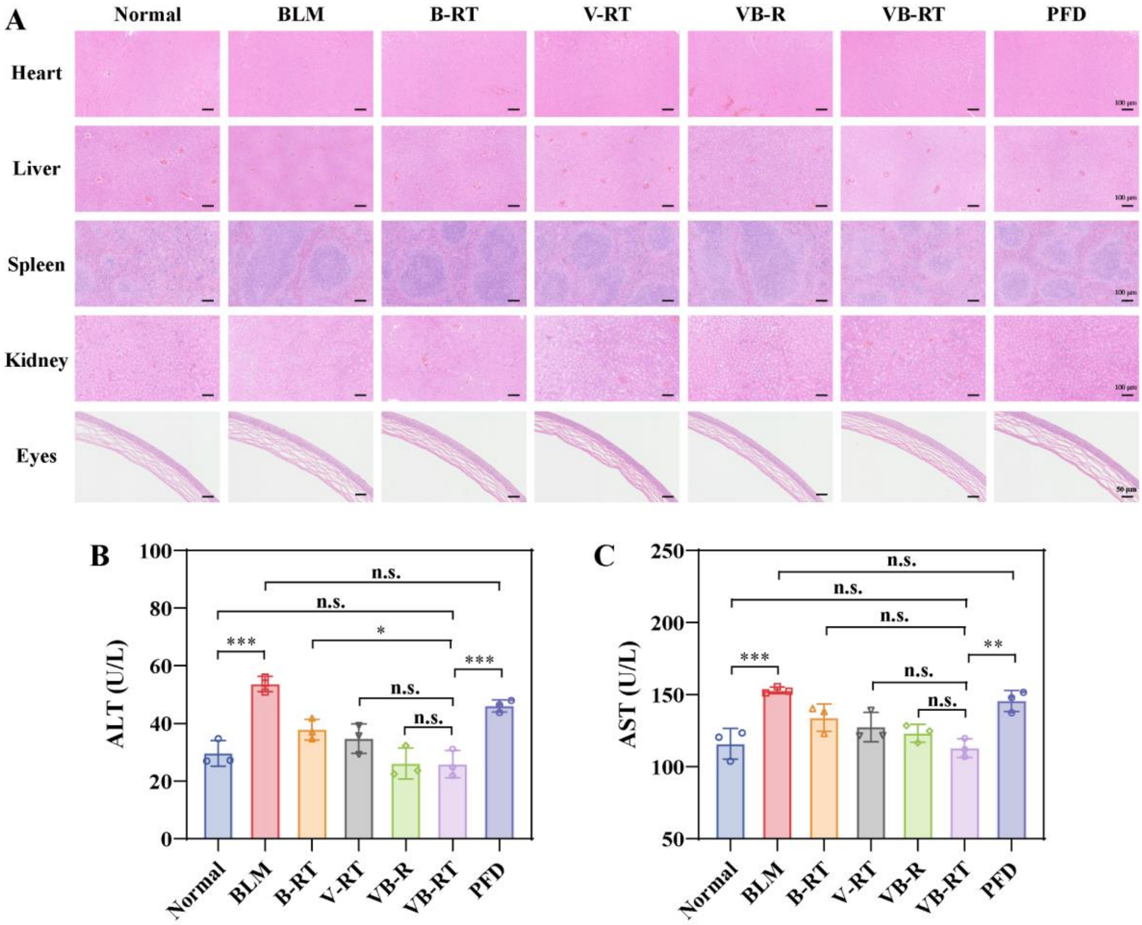

Figure S32. Evaluation of systemic toxicity and histological analysis in BLM-induced fibrotic mice treated with various formulations. A) H&E staining of heart, liver, spleen, kidney, and eye tissues to assess potential systemic toxicity following treatment. B-C) Serum levels of ALT (B) and AST (C) in fibrotic mice are treated with different formulations (n = 3). All data are

presented as the Mean ± SD. *p < 0.05, **p < 0.01, and ***p < 0.001. n.s., no significant difference.

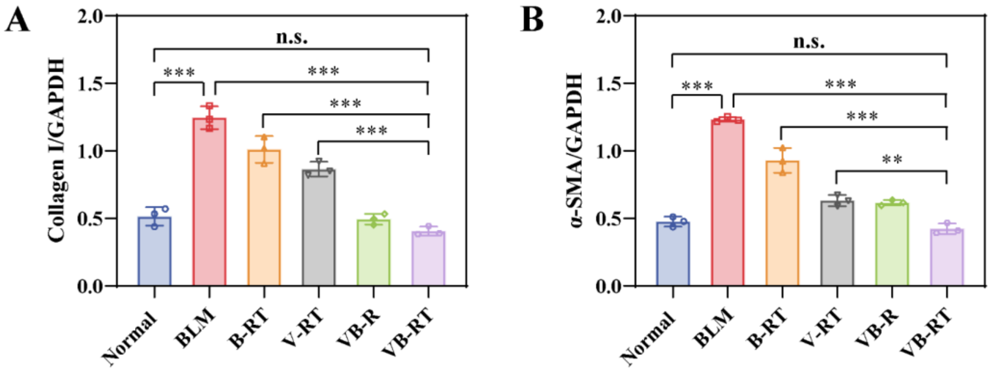

Figure S33. The Collagen I/GAPDH ratio (A) and α-SMA/GAPDH ratio (B) in mouse lung tissue analyzed using ImageJ (n = 3). All data are presented as the Mean ± SD (n = 3). **p < 0.01, and ***p < 0.001. n.s., no significant difference.

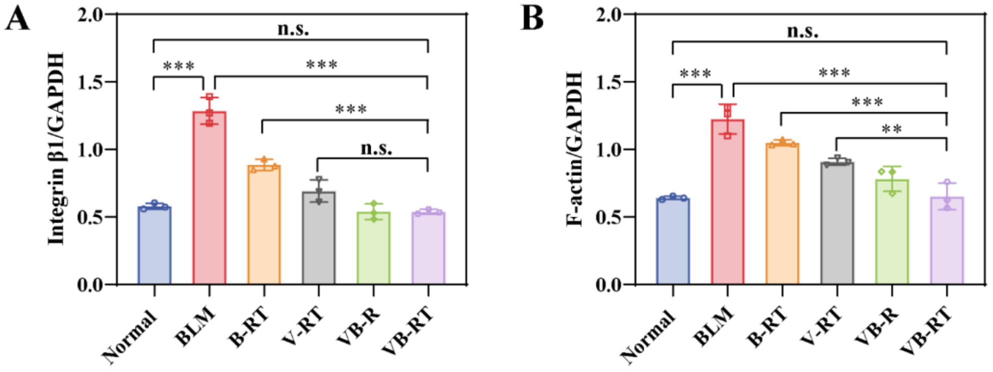

Figure S34. The integrin β1/GAPDH ratio (A) and F-actin/GAPDH ratio (B) in mouse lung tissue analyzed using ImageJ (n = 3). All data are presented as the Mean ± SD (n = 3). **p < 0.01, and ***p < 0.001. n.s., no significant difference.

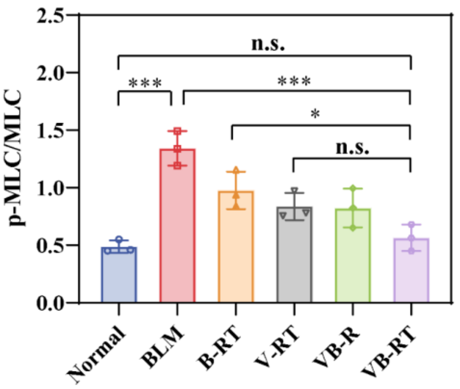

Figure S35. The p-MLC/MLC ratio in mouse lung tissue was analyzed using ImageJ (n = 3). All data are presented as the Mean ± SD (n = 3). *p < 0.05, ***p < 0.001. n.s., no significant difference.

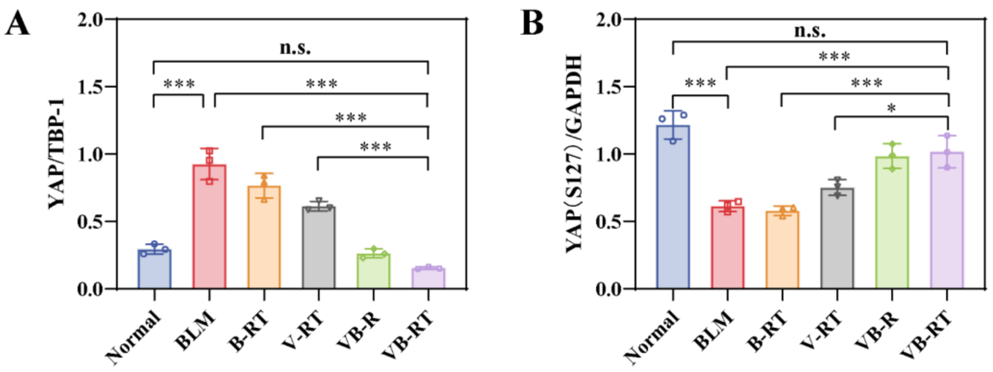

Figure S36. The YAP/TBP-1 ratio (A) and YAP (S127)/GAPDH ratio (B) in mouse lung tissue analyzed using ImageJ (n = 3). All data are presented as the Mean ± SD (n = 3). *p < 0.05, ***p < 0.001. n.s., no significant difference.

Table S1. Summary of antibody specifications.

Product	Supplier	Catalogue number	Dilution
rabbit anti-Vimentin	abcam	ab92547	1:1000
rabbit anti-TGF beta 1	Abclonal	A25313	1:1000
rabbit anti-VE-cadherin	Proteintech	27956-1-AP	1:1000
rabbit anti-CD31	abcam	ab28364	1:1000
rabbit anti-Phospho-Smad2-S250	Abclonal	AP1007	1:1000
rabbit anti-Smad3	Abclonal	A22133	1:1000
rabbit anti-Phospho-Myosin light chain (S20)	HuaBio	ER1913-46	1:1000
rabbit anti-monoclonal-MYL9 Recombinant	HuaBio	HA721766	1:1000
rabbit anti-alpha smooth muscle Actin	abcam	ab124964	1:10000
rabbit anti- Integrin-β1	Abclonal	A22599PM	1:2000
rabbit anti-polyclonal Collagen Type I	Proteintech	16495-1-AP	1:200
rabbit anti-YAP1	abcam	ab52771	1:5000
rabbit anti-YAP1 (phospho S127)	abcam	ab76252	1:10000
rabbit anti-FAP	abcam	ab218164	1:200
mouse anti-F-actin	abcam	ab130935	1:500
goat anti-rabbit IgG HRP-linked Ab	Beyotime	A0208	1:1000
goat anti-mouse IgG HRP-linked Ab	Beyotime	A0216	1:1000
rabbit anti-polyclonal GAPDH	abcam	ab9485	1:1000
rabbit anti-polyclonal TBP-1	HuaBio	HA500413	1:5000
goat anti-rabbit IgG Alexa Fluor 647	Beyotime	A0468	1:500
goat anti-mouse IgG (H+L) Coralite488	Proteintech	SA00013-1	1:500
Anti TGF-β1 LAP-D (R58)	Cosmo Bio	RIK-MA-R58	1:100

Table S2. qPCR primer sequences for *ALK5, FoxA2, Kdr*, *Tie2, Gapdh*.

Primer sequence	Forward primer	Reverse primer
ALK5	GCTGACATCTATGCAATGGGCTTA	AGGCAACTGGTAGTCTTCGTGGA
FoxA2	CCCTACGCCAACATGAACTCG	GTTCTGCCGGTAGAAAGGGA
Kdr	AAAATTGATCTTTGCTTGCTTACTG	CAGACCCTCATCACACTTTTCA
Tie2	ATGTGAACACCGAGGCTATTTG	CTTGACCTCGTGACAATCCA
Gapdh	GGACAATGGTGAAGGTCGGTGTGAAC	CAGCCGTGAGTGGAGTCATACTG
